# Evolutionary conservation in genes underlying human psychiatric disorders

**DOI:** 10.3389/fnhum.2014.00283

**Published:** 2014-05-06

**Authors:** Lisa M. Ogawa, Eric J. Vallender

**Affiliations:** Division of Neuroscience, New England Primate Research Center, Harvard Medical SchoolSouthborough, MA, USA

**Keywords:** schizophrenia, autism, *Homo sapiens*, adaptation

## Abstract

Many psychiatric diseases observed in humans have tenuous or absent analogs in other species. Most notable among these are schizophrenia and autism. One hypothesis has posited that these diseases have arisen as a consequence of human brain evolution, for example, that the same processes that led to advances in cognition, language, and executive function also resulted in novel diseases in humans when dysfunctional. Here, the molecular evolution of the protein-coding regions of genes associated with these and other psychiatric disorders are compared among species. Genes associated with psychiatric disorders are drawn from the literature and orthologous sequences are collected from eleven primate species (human, chimpanzee, bonobo, gorilla, orangutan, gibbon, macaque, baboon, marmoset, squirrel monkey, and galago) and 34 non-primate mammalian species. Evolutionary parameters, including d_N_/d_S_, are calculated for each gene and compared between disease classes and among species, focusing on humans and primates compared to other mammals, and on large-brained taxa (cetaceans, rhinoceros, walrus, bear, and elephant) compared to their small-brained sister species. Evidence of differential selection in humans to the exclusion of non-human primates was absent, however elevated d_N_/d_S_ was detected in catarrhines as a whole, as well as in cetaceans, possibly as part of a more general trend. Although this may suggest that protein changes associated with schizophrenia and autism are not a cost of the higher brain function found in humans, it may also point to insufficiencies in the study of these diseases including incomplete or inaccurate gene association lists and/or a greater role of regulatory changes or copy number variation. Through this work a better understanding of the molecular evolution of the human brain, the pathophysiology of disease, and the genetic basis of human psychiatric disease is gained.

## Introduction

Mental health disorders are common in the modern world, with lifetime prevalence ranging from 1 in 6 to 1 in 2 (Kessler et al., 2009). In the United States, roughly half of all Americans will suffer from a mental health disorder in their lifetime (Kessler et al., 2005); however understanding the causes of these diseases and developing efficacious treatments have been difficult. There are many reasons for these difficulties, but one particularly vexing issue for basic scientists has been in the difficulty in developing reliable and translational animal models (Nestler and Hyman, 2010; Razafsha et al., 2013).

Why animal models of neuropsychiatric disorders are challenging is multifaceted. No small part of this is due to the fact that the brain is incredibly complex and our understandings still incomplete. Another major issue is in identifying analogous behavioral phenotypes in animals; even seemingly straightforward behaviors like anxiety can be difficult to fully appreciate (Steimer, 2011). Before any of these considerations, however, lies the fundamental assumption that brains, their physiology, connectivity, and development, work the same across animals and that particular dysfunctions manifest similarly. The universal validity of this assumption however has been challenged, particularly as it relates to schizophrenia and autism.

Like other psychiatric diseases, schizophrenia and autism persist in society despite negative fitness effects that would put suffering individuals at an evolutionary disadvantage. Schizophrenia, which presents with characteristics ranging from delusions and hallucinations to impaired communication and social function, has a lifetime prevalence of roughly 0.5% (Goldner et al., 2002; Saha et al., 2005; Perala et al., 2007). Autism too shows characteristics similar to schizophrenia in its etiology and pathophysiology (De Lacy and King, 2013). Characterized by cognitive deficits ranging from impaired communication and social interaction to restricted and/or repetitive behaviors, autism spectrum disorders maintain a similar global prevalence of roughly 0.5%, skewed toward males (Elsabbagh et al., 2012).

Evidence supports an interpretation that the origins of schizophrenia and autism coincide with the divergence of modern humans (Crow, 1997; Horrobin, 1998; Burns, 2004). Despite proposed evolutionary advantages such as enhanced creativity in schizophrenia (Karlsson, 1984; Claridge et al., 1990; Post, 1994; Nettle, 2001), more often it is surmised that neurodevelopmental disease persists as a maladaptive by-product of changes in the brain that led to the human-specific traits of language (Crow, 1997), social cognition and interpersonal behavior (Burns, 2004, 2006), and increased brain size and changes in connectivity (Horrobin, 1998). Further support for the latter has been found in signatures of recent evolution in schizophrenia-associated genes in humans (Crespi et al., 2007), however this was a targeted study and since its publication new research has added to, and refined, gene-disease associations. Positive selection on genes associated with psychiatric disorders (Moalic et al., 2010) and disease (Fay, 2013) in general has also been previously identified, although whether this constitutes a general trend remains unclear.

A possible relationship between adaptation and common genetic variation in the etiology of schizophrenia and autism has been suggested since the first hereditary bases were observed (Farley, 1976). Conceptually this work has progressed in parallel with our understandings of complex genetic disease and molecular evolution. Initially much of the focus was on proteins with the belief that it was changes here that both drove the diseases and evolutionary processes. It was realized, at least in theory, early on that these dramatic changes in phenotype were more likely to be due to changes in regulatory mechanisms (King and Wilson, 1975). More recently, both communities have come to appreciate other sources of genetic variation. The role of copy number variation (CNV) in schizophrenia (Need et al., 2009; Stefansson et al., 2009; Raychaudhuri et al., 2010; Malhotra et al., 2011) and autism (Marshall et al., 2008; Bucan et al., 2009; Glessner et al., 2009; Pinto et al., 2010) has grown significantly and the fact that there is often significant overlap and balancing dosage effects between the two at the same CNV loci has been crucial to the development of the concept of a shared adaptive molecular etiology (Carroll and Owen, 2009; Crespi et al., 2010; Owen et al., 2011; Crespi and Crofts, 2012).

Here, we focus on the molecular evolution of protein-coding genes associated with schizophrenia, autism, and other neuropsychiatric diseases compared across mammalian species and among disease classes, with a focus on the primate (chimpanzee, bonobo, gorilla, orangutan, gibbon, macaque, baboon, marmoset, and squirrel monkey) and human lineages. Recent genome wide association studies (GWAS) have identified multiple genomic loci associated with autism (Ma et al., 2009; Wang et al., 2009; Weiss et al., 2009; Anney et al., 2010, 2012; Tsang et al., 2013) and Asperger syndrome (Salyakina et al., 2010), and schizophrenia (Fanous et al., 2012; Levinson et al., 2012; Aberg et al., 2013; Ripke et al., 2013). Using these datasets and similar meta-analyses in the literature that have identified genes implicated in neuropsychiatric disease it is possible to test whether mutations have occurred recently and uniquely in the evolution of humans, or whether similar changes are seen in other mammals. Approximated using ratios of non-synonymous (d_N_) to synonymous (d_S_) mutations in the protein-coding regions of genes, signatures of recent positive selection are evaluated. Evidence of differential selection on the human lineage in genes associated with the neurodevelopmental disorders to the exclusion of other neuropsychiatric diseases would support the hypothesis that these disorders are a cost of the higher brain function observed in humans.

## Materials and methods

### Genes associated with neurological and psychiatric disease

Genes associated with neurological and neuropsychiatric disease were drawn from the Genetic Association Database (GAD; http://geneticassociationdb.nih.gov/). This database is a publically-maintained repository of published associations between genes and complex diseases originating both from candidate gene studies as well as genome-wide association studies (Becker et al., 2004; Zhang et al., 2010). At the time this study was initiated the GAD included over 150,000 entries. This study focused on diseases associated with the brain and/or behavior and ultimately included 1920 gene-disease pairings, genes may be associated with more than one disease (Table 1). The nature of this approach is intentionally agnostic as to the exhaustiveness of these gene lists. Not only are these lists not likely to be complete, it is entirely possible that false positives are included. Rather than force an attempt at an unobtainable ideal, all putative positive associations have been included. The implications of this approach and its relationship to the results obtained are discussed herein.

**Table 1:** **Number of genes from Genetic Association Database by disease**.

**Disease**	**Number of genes**
Addiction	334
Alzheimer disease	254
Anxiety	18
Attention Deficit Hyperactivity Disorder (ADHD)	183
Autism/Asperger	34
Bipolar disorder	227
Depression	85
Eating disorder	14
Migraine	19
Parkinson disease	194
Schizophrenia	199
Stroke	341

Because of previous discussions in the literature, special attention was given to genes associated with schizophrenia and autism. For these diseases more complete and highly curated lists of associated genes are available.

The Schizophrenia Gene Resource (SzGR) is likewise a repository of more than 7500 genes showing links with schizophrenia (Jia et al., 2010). This includes not only genes associated with the disease but also those that undergo expression changes in cases and gene network and pathway analyses integrating a wide variety of data. Here, five subsets of these data are used for analysis: a core list of 38 genes (Ross et al., 2006; Allen et al., 2008), a more inclusive list of 278 protein-coding genes associated with schizophrenia (Sun et al., 2008), a prioritized list of 75 associated genes by combined odds ratio (COR) (Sun et al., 2008), or gene prioritization (Sun et al., 2009) lists derived from two linkage meta-analyses: (Lewis et al., 2003), 160 genes, or (Ng et al., 2009), 173 genes.

The Simons Foundation Autism Research Initiative (SFARI) maintains a database of genes associated with autism spectrum disorders (Basu et al., 2009). As of January 2014 this included more than 500 genes, and more than half of these have been scored by an expert advisory panel for the strength of their evidence (Abrahams et al., 2013). This study uses 516 genes including 303 with scores available.

A list of all genes used in this study and their categorization can be found in supplemental materials (Table S1).

### Ortholog identification

Forty-five species were used for the identification of orthologs (Table 2): eleven primates including humans, 31 non-primate eutherian mammals, two marsupials, and one monotreme. Protein-coding sequences for each species were drawn from RefSeq (Pruitt et al., 2014). Using BLAST (Boratyn et al., 2013), best hits were identified for each species using the human sequence and the universe of species-specific RefSeq target sequences (Table S2). Putative orthologs with d_S_ rates greater than 4 standard deviations from the median for the human-species pairwise comparison were flagged for manual curation and removed from the data set as warranted (Vallender, 2009).

**Table 2:** **Species used in the study**.

	**Species**	**Common name**
Monotremes	*Ornithorhynchus anatinus*	Platypus
Marsupials	*Monodelphis domestica*	Gray short-tailed opossum
	*Sarcophilus harrisii*	Tasmanian devil
Eutheria		
Xenarthra	*Dasypus novemcinctus*	Nine-banded armadillo
Afrotheria	*Trichechus manatus latirostris*	Florida manatee
	*Echinops telfairi*	Small Madagascar hedgehog
	*Loxodonta africana*	African savanna elephant
Laurasiatheria		
Eulipotyphla	*Condylura cristata*	Star-nosed mole
	*Sorex araneus*	European shrew
Perissodactyla	*Equus caballus*	Horse
	*Ceratotherium simum simum*	Southern white rhinoceros
Cetartiodactyla	*Sus scrofa*	Pig
	*Bos taurus*	Cow
	*Ovis aries*	Sheep
	*Orcinus orca*	Killer whale
	*Tursiops truncatus*	Bottlenosed dolphin
Carnivora	*Felis catus*	Cat
	*Canis lupus familiaris*	Dog
	*Mustela putorius furo*	Ferret
	*Ailuropoda melanoleuca*	Giant panda
	*Odobenus rosmarus divergens*	Pacific walrus
Euarchontoglires		
Lagomorpha	*Oryctolagus cuniculus*	Rabbit
	*Ochotona princeps*	American pika
Rodentia	*Chinchilla lanigera*	Long-tailed chinchilla
	*Heterocephalus glaber*	Naked mole-rat
	*Octodon degus*	Degu
	*Cavia porcellus*	Guinea pig
	*Jaculus jaculus*	Lesser Egyptian jerboa
	*Ictidomys tridecemlineatus*	Thirteen-lined ground squirrel
	*Microtus ochrogaster*	Prairie vole
	*Mesocricetus auratus*	Golden hamster
	*Cricetulus griseus*	Chinese hamster
	*Rattus norvegicus*	Rat
	*Mus musculus*	Mouse
Primates	*Otolemur garnettii*	Small-eared galago
	*Saimiri boliviensis boliviensis*	Bolivian squirrel monkey
	*Callithrix jacchus*	White-tufted-ear marmoset
	*Macaca mulatta*	Rhesus macaque
	*Papio anubis*	Olive baboon
	*Nomascus leucogenys*	Northern white-cheeked gibbon
	*Pongo abelii*	Sumatran orangutan
	*Gorilla gorilla gorilla*	Western lowland gorilla
	*Pan troglodytes*	Chimpanzee
	*Pan paniscus*	Bonobo (pygmy chimpanzee)
	*Homo sapiens*	Human

### Evolutionary calculations

**Figure 1:**
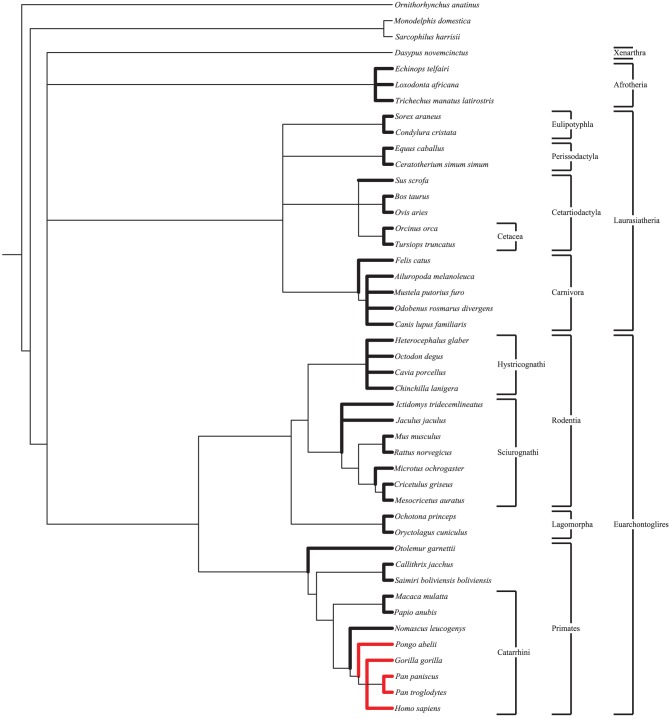
**Species tree**. Species tree, generated by NCBI Taxonomy Browser, used as a guide in PAML for the calculation of d_N_ and d_S_ values under the free ratio model. Lineages presented in this study are bolded. Great ape species are shown in red for ease of comparison. Notable clades discussed herein are shown.

Following ortholog identification nucleotide sequences were translated into proteins and aligned using CLUSTALW (Larkin et al., 2007). These aligned protein sequences were then used to align the original nucleotide sequences in frame. Using a species tree (Figure 1) generated by the NCBI Taxonomy Browser (NCBI Resource Coordinators, 2014) as a guide, PAML (Yang, 2007) was used to calculate d_N_ and d_S_ for all lineages. Default values within the PAML control files were used. For each gene four models were run: a single d_N_/d_S_ for all branches, a branch model comparing catarrhine branches to all others, a branch model comparing catarrhine and cetacean branches to all others, and a free ratio model (Table S3). Results were not significantly different when a more nuanced tree (Meredith et al., 2011), or when the Nei and Gojobori method was used (Nei and Gojobori, 1986). To calculate average d_N_/d_S_ for groups of genes, such as those associated with a specific disorder, the average of d_N_ divided by the average of d_S_, *d_N_*/*d_S_*, was used rather than the average of d_N_/d_S_, (*d_N_/d_S_*), after previous work (Vallender and Lahn, 2007). This method of calculation better represents evolutionary constraint on the group of genes particularly in short lineages up against the zero bound for d_S_ where stochastic noise is large relative to the expected value.

### Statistical calculations

Statistical comparisons were made using One-Way ANOVA between all lineage pairs with Bonferroni correction for multiple tests. This method results in a conservative estimation of significance, but one without any *a priori* biases. Summaries of statistical significance are offered in the text, but readers wanting additional information are referred to Supplemental Tables 4–6.

## Results

This study sought to understand the evolutionary conservation among genes associated with mental health disorders. It made use of genomes from 42 eutherians and 3 monotreme and marsupial outgroups. For each gene, orthologs were identified where possible and the protein sequences aligned. Evolutionary parameters, including mutation rates at synonymous (d_S_) and non-synonymous sites (d_N_), and measures of evolutionary constraint (d_N_/d_S_) were calculated for each branch. Prior to any attempt at interpreting the data, two issues of importance need to be addressed: the identification of genes under study and the lineages under focus.

A pervasive difficulty in any attempt to study the genetics underlying a particular disease is first in developing a list of genes responsible for the disease. This is already challenging for complex polygenic diseases where phenotypic delineations are clear. For mental health disorders where symptomology and diagnosis are more problematic, it is a significant issue. Lists of genes associated with these diseases are almost certainly incomplete and likely will see disagreement even among experts. An advantage of using a literature-derived aggregator like the Genetic Association Database (GAD) is that it is unbiased, even at the cost of potentially including what ultimately may prove to be false positives.

The evolutionary constraint for the terminal branches of each of the eutherians are compared with the exception of the armadillo (*Dasypus novemcinctus*), the only species lacking an intra-ordinal comparison. Afrotherian phylogenetics and species representation poses difficulties as well (Seiffert, 2007) complicating direct comparisons among those species pairs and those of Boreoeutheria. Internal branches likewise proved difficult to analyze due in part to missing orthologs causing the collapse of multiple branches into one. Internal branches are also quantitatively different than terminal branches; terminal branches not only are more likely to include sequencing artifacts, but they are also likely, especially in situations where few exemplars were used in the genome building process, to include polymorphisms destined to be eliminated by selection (genetic load). Because these events are biased toward non-synonymous mutations, they artificially raise the apparent d_N_/d_S_. While this pattern of effects is the same across genes and gene categories, it is not necessarily directly comparable across species making it important that these issues be taken into account when interpreting findings.

**Figure 2:**
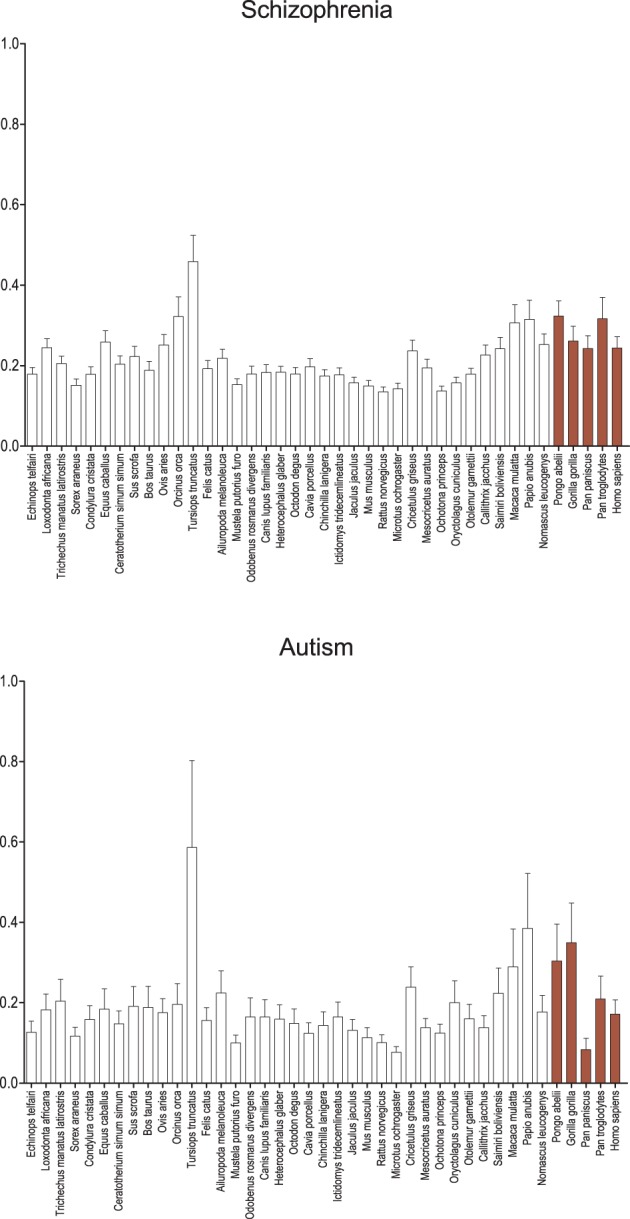
**Average d_N_/d_S_ across taxa of genes associated with schizophrenia and autism**. Average d_N_/d_S_ of genes positively associated with schizophrenia (top) and autism (bottom) based on positive gene-disease associations collated by the Genetic Association Database (GAD) are depicted. Average d_N_/d_S_ ratios suggest changes in selective regime in catarrhines and dophin in genes associated with these neurodevelopmental diseases. Pairwise significance values are shown in Supplemental Table 4.

While genes associated with schizophrenia and autism based on positive associations in the GAD (Figure 2) reveal no significant signal of positive selection on the human branch relative to apes or other primates, catarrhines in general show higher average d_N_/d_S_ when compared to other mammals, with the exception of the cetaceans (*Tursiops truncatus* and *Orcinus orca*). Among the schizophrenia-associated genes, catarrhines tend to show a significant difference in d_N_/d_S_ relative to most sciurognaths (*p* < 0.05), with similar although non-significant trends when compared to the other rodents, lagomorphs, carnivores, and insectivores (Eulipotyphla). Among the autism-associated genes, results are trending in a similar direction, however the signal contains more noise likely due to the fewer number of genes analyzed. The cetaceans, especially the dolphin branch, show significantly different evolutionary constraint on genes associated with schizophrenia and autism, with a greater average d_N_/d_S_ than almost all other mammals.

**Figure 3:**
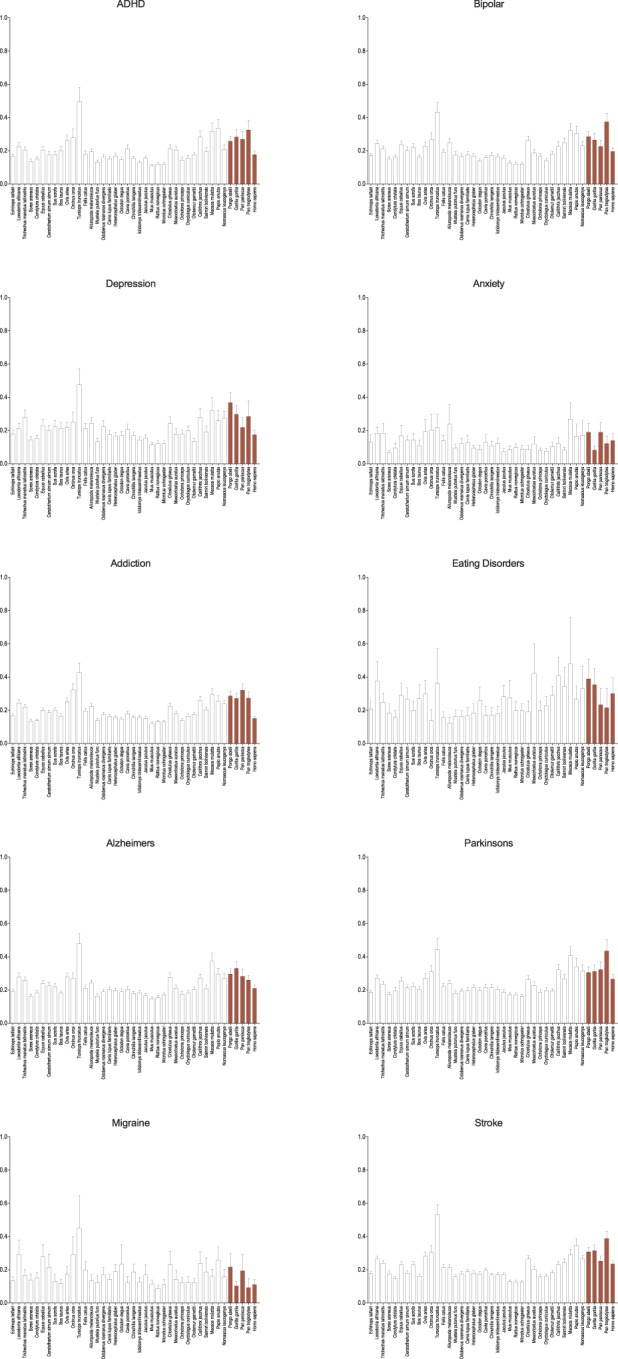
**Average d_N_/d_S_ across taxa of genes associated with other neurological diseases and controls**. Average d_N_/d_S_ of genes positively associated with other neurological diseases (ADHD, bipolar disorder, depression, anxiety, addiction, eating disorders, Alzheimers, and Parkinsons, migraine) and control (stroke) based on positive gene-disease associations in the Genetic Association Database (GAD) are depicted. Average d_N_/d_S_ ratios reveal a similar pattern of evolution in catarrhines and dophin as was seen with schizophrenia and autism, to the exclusion of anxiety, eating disorder, and migraine which depict broad evolutionary conservation across all evaluated mammalian taxa. Pairwise significance values are shown in Supplemental Table 4.

Among other neurological disorders similar results are seen (Figure 3). The general pattern observed in genes associated with schizophrenia are seen in ADHD, bipolar disorder, addiction, and the neurodegenerative disorders (Alzheimers and Parkinsons), though as with autism the difference is less. Only in genes associated with anxiety, eating disorders, and migraine is this broad pattern not seen and in depression it is greatly diminished. With each of these categories it may simply be the result of small numbers of genes, but genes associated with anxiety and migraine are noticeably and dramatically more conserved across all species suggesting perhaps a different selective regime. Despite their neurological effects, genes associated with stroke are largely vascularly, not neuronally, expressed, and therefore serve as a useful control for the other neurological diseases. While muted, it too shows similar patterns as seen in schizophrenia and the majority of the other mental health disorders.

As previously noted, one potential difficulty in these analyses is in the identification of specific genes associated with disease. Focusing specifically on schizophrenia and autism, the two diseases hypothesized to be associated with the evolution of the human brain, additional gene lists were sought out to confirm and develop these findings. The Schizophrenia Gene Resource (SzGR, Jia et al., 2010) and Simons Foundation Autism Research Initiative Gene (SFARI, Basu et al., 2009) both contain gene lists maintained and curated by disease experts and are therefore potentially more complete and accurate.

**Figure 4:**
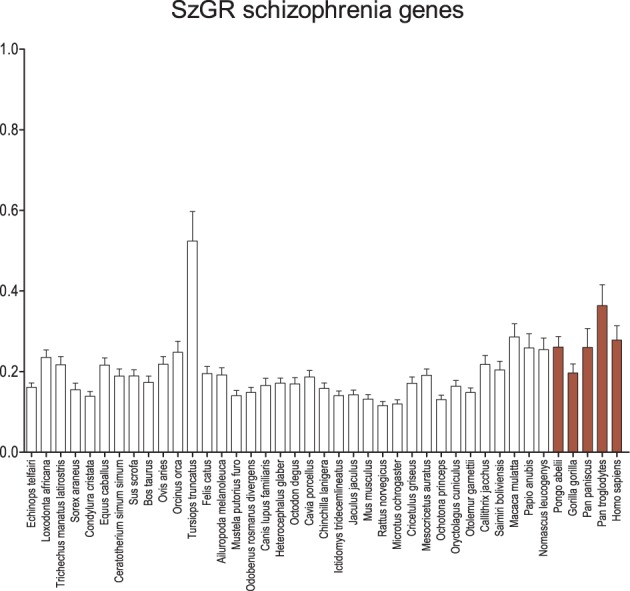
**Average d_N_/d_S_ across taxa of SzGR schizophrenia-associated genes**. Average d_N_/d_S_ of genes positively associated with schizophrenia based on associations collected in the Schizophrenia Gene Resource (SzGR). Average d_N_/d_S_ ratios suggest changes in selective regime in catarrhines and dophin similar to the GAD gene set. Pairwise significance values are shown in Supplemental Table 5.

When the genes listed by SzGR as associated with schizophrenia are examined (Figure 4), an almost identical pattern emerges compared to the GAD genes. Again, the dolphin shows significantly greater d_N_/d_S_ values than other mammals and catarrhine d_N_/d_S_ values are elevated, reaching significance in some comparisons. Interestingly, these patterns are ensmalled when attempts are made to prioritize the genes and identify those that are most important (Figure S1). It is possible that this results from the approaches taken to prioritization, but it also may suggest that those genes more central to the disease have a distinct signature of conservation.

**Figure 5:**
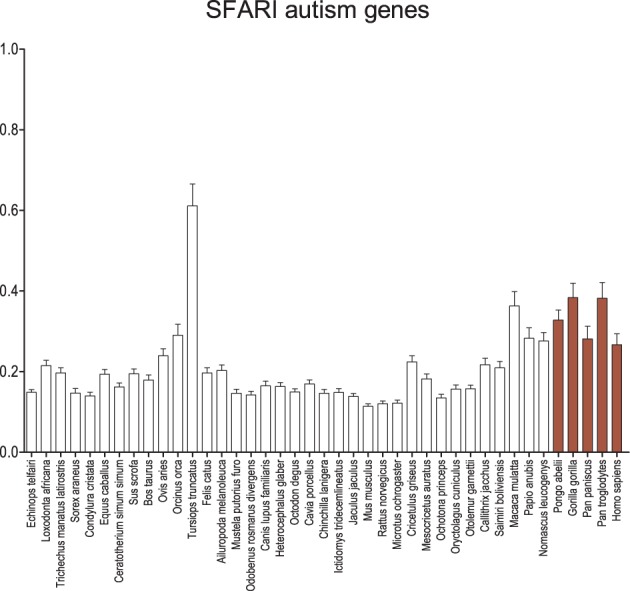
**Average d_N_/d_S_ across taxa of SFARI autism-associated genes**. Average d_N_/d_S_ of genes positively associated with autism based on associations collected in Simons Foundation Autism Research Initiative Gene (SFARI). Average d_N_/d_S_ ratios suggest changes in selective regime in catarrhines and dophin similar to what is observed for schizophrenia gene sets but a stronger pattern than observed in the GAD autism gene set. Pairwise significance values are shown in Supplemental Table 6.

Using the SFARI dataset for autism genes, a much more robust recapitulation of the GAD pattern is observed (Figure 5). Although the average d_N_/d_S_ on the human branch remains equal to or less than the other apes and old world monkeys, overall the catarrhines, including humans, maintain a significantly greater average d_N_/d_S_ when compared to the insectivores (*p* < 0.001), some carnivores (*p* < 0.05), most rodents (*p* < 0.05), and the lagomorphs (*p* < 0.01). Genes for syndromic and non-syndromic autism were also evaluated separately with no difference in the overall result pattern (Figure S2), except perhaps a greater signal and larger error among the apes and old world monkeys in syndromic autism. Analyses were also performed on scored datasets (Figure S3), where increasing level of confidence in genes associated with autism revealed few differences in the overall results.

## Discussion

Our understanding of neurodevelopmental disorders has led to the hypothesis that diseases like schizophrenia and autism are uniquely human. The implication stemming from this idea is that identifying the genes underlying the diseases will also identify the genetic changes that have led, at least in part, to the evolution of the human brain. Through evaluation of the molecular evolution of genes associated with schizophrenia and autism, it is possible to detect signatures of positive selection. In this study, however, elevated d_N_/d_S_ values in genes associated with these diseases were not restricted to the human branch, and instead were seen not only in humans and apes, but in the old world monkeys and dolphins as well.

Elevated d_N_/d_S_ values by themselves are not proof of positive selection, because relaxation of selective constraint would produce similar results. Positive selection on a gene also can be obscured by differences in selective pressures across time or by differences in pressures in different protein regions. Furthermore, here, when considering classes of genes, an additional factor is present; some genes within a class may be under positive selection while others may not. Nevertheless, the very nature of the genes and their association with significant diseases makes it unlikely that they are under relaxed constraint. It remains that these effects could be of demographic origins, resulting from inefficient selective pressures due to reduced effective population sizes or lineage lengths. This is given credence in part because a similar, if weakened, pattern of selection was also observed in non-neurodevelopmental diseases with few differences distinguishing schizophrenia and autism from ADHD, bipolar disease, the neurodegenerative diseases, addiction, and the control pathology of stroke. Because these latter diseases have not been suggested to be a unique consequence of human evolution there is less reason to expect selective pressures leading to the same pattern. Indeed the conservation observed in the genes associated with migraine and anxiety is much more similar to the expected control, with strong conservation across all species. These findings also broadly follow the expectations of the nearly-neutral model of molecular evolution (Ohta, 1995; Akashi et al., 2012).

Genome quality may also be a partial cause of this effect. Certainly poor quality genomes would be expected to show higher d_N_/d_S_ as errors would fall neutrally on genes. Even without sequencing errors, genomes constructed using few individuals can appear to have artificially higher d_N_/d_S_ values because of an inability to exclude slightly deleterious mutations. The human genome is by far the best studied and its consensus is based on a much greater number of individuals, therefore the apparent genetic load on this branch is likely less than the other mammals, dampening the signal of positive selection relative to the other taxa. Many, though not all, of the other species for which genomes are available use fewer individuals. Additionally several of the species for which genomes are available have recent histories of artificial selection, whether through domestication (pig, cow, sheep, cat, and dog) or laboratorization (mouse and rat), that may have fixed some level of genetic load.

Regardless, the signals that are observed are not unique to humans; rather we see similar patterns across catarrhines and cetaceans. This may suggest that any changes in selective regime on genes associated with these diseases is a result of a shared elaboration; in fact, toothed cetaceans and primates are known to share similar patterns of molecular evolution (McGowen et al., 2012), relaxation of constraint on encephalization, and an overall greater brain to body mass ratio when compared to other mammalian taxa (Boddy et al., 2012), a phenotype potentially attributed to enhanced social behavior (Connor, 2007). Likewise, positive selection on genes associated with nervous system processes including synaptic plasticity, sleep, and intellectural disabilities including schizophrenia have also been previously detected in dolphins (McGowen et al., 2012). Notably, however, other large-brained taxa evaluated in this study, including elephants, bears, and rhinoceroses, did not show similar differences, suggesting that the changes observed are not simply the result of changes in brain size. Furthermore consistent differences in evolutionary constraint are seen in catarrhines relative to the sciurognaths, which include laboratory rodents *Mus musculus* and *Rattus norvegicus*. This result might suggest increased caution when using these animals as models of neuropsychiatric disease.

Thus although these results suggest that selection on the genes associated with schizophrenia and autism is not associated with humans alone, the tenuousness or absence of neurodevelopmental disease analogs in other species remains an important consideration, and therefore may point at potential limitations in study design, including the gene-disease association lists themselves. Given the polygenic nature of neuropsychiatric diseases, the broad spectrum of phenotypes that represent disease, and the continued limited understanding of the molecular mechanism by which diseases are triggered, genes with weak, or unconfirmed associations included in the analysis have the potential to mask a signal of positive selection. Furthermore, if genes strongly associated with the disease have not yet been identified, then a signal of positive selection may again be weakened. This is supported at least in part by the changes seen in genes associated with autism when the SFARI gene list is used.

Lastly, it is also possible that a signal for positive selection on the human branch was missed in this analysis because neurodevelopmental disorders result not from changes in the genes themselves, but instead from changes in regulatory regions or variation in copy number. Although positive selection in protein-coding regions of genes associated with language (FOXP2, Enard et al., 2002) and brain size (MCPH1, Evans et al., 2004a; Wang and Su, 2004); ASPM, (Zhang, 2003; Evans et al., 2004b; Kouprina et al., 2004) have been found in humans, this may be because these changes are easier to identify at present. Temporal or spatial changes in expression patterns or changes in the amplitude of expression of the genes may show differences across mammals and are perhaps more likely to be uniquely different in humans (King and Wilson, 1975). Haygood et al. (2007), for instance, found a human-specific signature of positive selection in promoter regions of genes associated with neural development and function as well as in neurodegenerative diseases. Studies of structural variation have also found indications of brain-related evolutionary effects in humans (Cooper et al., 2007); and copy number variation of the DUF1220 domain has been associated with both autism severity (Davis et al., 2014) and brain-size evolution in primates (Popesco et al., 2006; Dumas et al., 2012). It may be that while the proteins associated with schizophrenia and autism are indeed conserved between humans and other mammals, their expression has diverged.

One implication of these results may be that when we study neurodevelopmental disorders from an evolutionary standpoint we should not be focusing on proteins. Rather, those aspects of the disease that may represent an adaptive evolutionary response are found in other genetic moieties, either in regulatory regions or in structural and copy number variation. It is perhaps worth considering if this represents a unique situation in the neurodevelopmental diseases or if it is simply a reflection of more general patterns of adaptive evolution. It is also perhaps relevant to our understandings of the neurodevelopmental diseases themselves. If the evolutionary changes associated with the disease are to be found outside of proteins, then are we also more likely to find treatments or cures there?

Although these data suggest that genes associated with neurodevelopmental disease are not evolving uniquely in humans, the study is potentially limited by the completeness and accuracy of gene-disease association lists available and by the methodology of the study that focuses on protein-coding regions. Rather than refute the hypothesis, however, it may point toward a greater importance for changes in expression, in promoters and/or transcription factor binding sites, or in epigenetic markings in the lineage leading to humans.

### Conflict of interest statement

The authors declare that the research was conducted in the absence of any commercial or financial relationships that could be construed as a potential conflict of interest.

## Supplementary material

Figure S1 **Average d_N_/d_S_ across taxa using subsets of the SzGR schizophrenia-associated genes**. Average d_N_/d_S_ of genes positively associated with schizophrenia based on associations collected in the Schizophrenia Gene Resource (SzGR), subdivided by groups by various attempts at prioritization. Pairwise significance values are shown in Supplemental Table 4.

Figure S2 **Average d_N_/d_S_ across taxa comparing SFARI syndromic vs. non-syndromic autism-associated genes**. Average d_N_/d_S_ of genes positively associated with autism based on associations collected in Simons Foundation Autism Research Initiative Gene (SFARI) subdivided into genes associated with syndromic and non-syndromic autism. Pairwise significance values are shown in Supplemental Table 5.

Figure S3 **Average d_N_/d_S_ across taxa comparing SFARI scored autism-associated genes**. Average d_N_/d_S_ of genes positively associated with autism based on associations collected in Simons Foundation Autism Research Initiative Gene (SFARI) subdivided by the scoring system used by SFARI Gene: all scored genes, genes scored with hypothesized association (5+), genes scored with at least minimal evidence (4+), and genes scored with at least suggestive evidence (3+). Pairwise significance values are shown in Supplemental Table 6.
