# Supplementary material for: Evolutionary conservation in genes underlying human psychiatric disorders
Source: Front Hum Neurosci. 2014 May 6;8:283. doi: 10.3389/fnhum.2014.00283 (PMC4018557; doi:10.3389/fnhum.2014.00283)
Supplement: Figure S2 — Average dN/dS across taxa comparing SFARI syndromic vs. non-syndromic autism-associated genes. Average dN/dS of genes positively associated with autism based on associations collected in Simons Foundation Autism Research Initiative Gene (SFARI) subdivided into genes associated with syndromic and non-syndromic autism. Pairwise significance values are shown in Supplemental Table 5. [file DataSheet6.PDF]

Figure 5. Simons Foundation Autism Research Initiative Gene

	Loxodonta_africana	Trichechus_manatus_latirostris	Sorex_araneus	Condylura_cristata	Equus_caballus	Ceratotherium_simum_simum	Sus_scrofa	Bos_taurus	Ovis_aries	Orcinus_orca	Tursiops_truncatus	Felis_catus	Ailuropoda_melanoleuca	Mustela_putorius_furo	Odobenus_rosmarus_divergens	Canis_lupus_familiaris	Heterocephalus_glaber	Octodon_degus	Cavia_porcellus	Chinchilla_lanigera	Ictidomys_tridecemlineatus	Jaculus_jaculus	Mus_musculus	Rattus_norvegicus	Microtus_ochrogaster	Cricetulus_griseus	Mesocricetus_auratus	Ochotona_princeps	Oryctolagus_cuniculus	Otolemur_garnettii	Callithrix_jacchus	Saimiri_boliviensis	Macaca_mulatta	Papio_anubis	Nomascus_leucogenys	Pongo_abelii	Gorilla_gorilla	Pan_paniscus	Pan_troglodytes	Homo_sapiens
Echinops_telfairi	ns	ns	ns	ns	ns	ns	ns	ns	ns	****	****	ns	ns	ns	ns	ns	ns	ns	ns	ns	ns	ns	ns	ns	ns	ns	ns	ns	ns	ns	ns	ns	****	***	***	****	****	***	****	**
Loxodonta_africana		ns	ns	ns	ns	ns	ns	ns	ns	ns	****	ns	ns	ns	ns	ns	ns	ns	ns	ns	ns	ns	*	ns	ns	ns	ns	ns	ns	ns	ns	ns	****	ns	ns	*	****	ns	****	ns
Trichechus_manatus_latirostris			ns	ns	ns	ns	ns	ns	ns	ns	****	ns	ns	ns	ns	ns	ns	ns	ns	ns	ns	ns	ns	ns	ns	ns	ns	ns	ns	ns	ns	ns	****	ns	ns	***	****	ns	****	ns
Sorex_araneus				ns	ns	ns	ns	ns	ns	****	****	ns	ns	ns	ns	ns	ns	ns	ns	ns	ns	ns	ns	ns	ns	ns	ns	ns	ns	ns	ns	ns	****	****	***	****	****	***	****	**
Condylura_cristata					ns	ns	ns	ns	ns	****	****	ns	ns	ns	ns	ns	ns	ns	ns	ns	ns	ns	ns	ns	ns	ns	ns	ns	ns	ns	ns	ns	****	****	****	****	****	****	****	***
Equus_caballus						ns	ns	ns	ns	ns	****	ns	ns	ns	ns	ns	ns	ns	ns	ns	ns	ns	ns	ns	ns	ns	ns	ns	ns	ns	ns	ns	****	ns	ns	***	****	ns	****	ns
Ceratotherium_simum_simum							ns	ns	ns	***	****	ns	ns	ns	ns	ns	ns	ns	ns	ns	ns	ns	ns	ns	ns	ns	ns	ns	ns	ns	ns	ns	****	**	**	****	****	**	****	*
Sus_scrofa								ns	ns	ns	****	ns	ns	ns	ns	ns	ns	ns	ns	ns	ns	ns	ns	ns	ns	ns	ns	ns	ns	ns	ns	ns	****	ns	ns	***	****	ns	****	ns
Bos_taurus									ns	*	****	ns	ns	ns	ns	ns	ns	ns	ns	ns	ns	ns	ns	ns	ns	ns	ns	ns	ns	ns	ns	ns	****	*	ns	****	****	ns	****	ns
Ovis_aries										ns	****	ns	ns	ns	ns	ns	ns	ns	ns	ns	ns	*	***	**	**	ns	ns	*	ns	ns	ns	ns	**	ns	ns	ns	****	ns	****	ns
Orcinus_orca											****	ns	ns	****	****	***	***	****	**	****	****	****	****	****	****	ns	*	****	***	***	ns	ns	ns	ns	ns	ns	ns	ns	ns	ns
Tursiops_truncatus												****	****	****	****	****	****	****	****	****	****	****	****	****	****	****	****	****	****	****	****	****	****	****	****	****	****	****	****	****
Felis_catus													ns	ns	ns	ns	ns	ns	ns	ns	ns	ns	ns	ns	ns	ns	ns	ns	ns	ns	ns	ns	****	ns	ns	***	****	ns	****	ns
Ailuropoda_melanoleuca														ns	ns	ns	ns	ns	ns	ns	ns	ns	ns	ns	ns	ns	ns	ns	ns	ns	ns	ns	****	ns	ns	***	****	ns	****	ns
Mustela_putorius_furo															ns	ns	ns	ns	ns	ns	ns	ns	ns	ns	ns	ns	ns	ns	ns	ns	ns	ns	****	****	***	****	****	****	****	**
Odobenus_rosmarus_divergens																ns	ns	ns	ns	ns	ns	ns	ns	ns	ns	ns	ns	ns	ns	ns	ns	ns	****	****	****	****	****	****	****	***
Canis_lupus_familiaris																	ns	ns	ns	ns	ns	ns	ns	ns	ns	ns	ns	ns	ns	ns	ns	ns	****	**	**	****	****	**	****	*
Heterocephalus_glaber																		ns	ns	ns	ns	ns	ns	ns	ns	ns	ns	ns	ns	ns	ns	ns	****	**	**	****	****	**	****	*
Octodon_degus																			ns	ns	ns	ns	ns	ns	ns	ns	ns	ns	ns	ns	ns	ns	****	***	***	****	****	***	****	**
Cavia_porcellus																				ns	ns	ns	ns	ns	ns	ns	ns	ns	ns	ns	ns	ns	****	**	*	****	****	*	****	ns
Chinchilla_lanigera																					ns	ns	ns	ns	ns	ns	ns	ns	ns	ns	ns	ns	****	****	***	****	****	***	****	**
Ictidomys_tridecemlineatus																						ns	ns	ns	ns	ns	ns	ns	ns	ns	ns	ns	****	****	***	****	****	***	****	**
Jaculus_jaculus																							ns	ns	ns	ns	ns	ns	ns	ns	ns	ns	****	****	****	****	****	****	****	***
Mus_musculus																								ns	ns	**	ns	ns	ns	ns	*	ns	****	****	****	****	****	****	****	****
Rattus_norvegicus																									ns	*	ns	ns	ns	ns	ns	ns	****	****	****	****	****	****	****	****
Microtus_ochrogaster																										*	ns	ns	ns	ns	ns	ns	****	****	****	****	****	****	****	****
Cricetulus_griseus																											ns	ns	ns	ns	ns	ns	****	ns	ns	ns	****	ns	****	ns
Mesocricetus_auratus																												ns	ns	ns	ns	ns	****	ns	ns	****	****	ns	****	ns
Ochotona_princeps																													ns	ns	ns	ns	****	****	****	****	****	****	****	***
Oryctolagus_cuniculus																														ns	ns	ns	****	**	**	****	****	**	****	*
Otolemur_garnettii																															ns	ns	****	***	**	****	****	***	****	*
Callithrix_jacchus																																ns	****	ns	ns	*	****	ns	****	ns
Saimiri_boliviensis																																	****	ns	ns	**	****	ns	****	ns
Macaca_mulatta																																		ns	ns	ns	ns	ns	ns	ns
Papio_anubis																																			ns	ns	ns	ns	ns	ns
Nomascus_leucogenys																																				ns	*	ns	*	ns
Pongo_abelii																																					ns	ns	ns	ns
Gorilla_gorilla																																						ns	ns	**
Pan_paniscus																																							ns	ns
Pan_troglodytes																																								**

Figure S2A. SFARI - Syndromic

	Loxodonta_africana	Trichechus_manatus_latirostris	Sorex_araneus	Condylura_cristata	Equus_caballus	Ceratotherium_simum_simum	Sus_scrofa	Bos_taurus	Ovis_aries	Orcinus_orca	Tursiops_truncatus	Felis_catus	Ailuropoda_melanoleuca	Mustela_putorius_furo	Odobenus_rosmarus_divergens	Canis_lupus_familiaris	Heterocephalus_glaber	Octodon_degus	Cavia_porcellus	Chinchilla_lanigera	Ictidomys_tridecemlineatus	Jaculus_jaculus	Mus_musculus	Rattus_norvegicus	Microtus_ochrogaster	Cricetulus_griseus	Mesocricetus_auratus	Ochotona_princeps	Oryctolagus_cuniculus	Otolemur_garnettii	Callithrix_jacchus	Saimiri_boliviensis	Macaca_mulatta	Papio_anubis	Nomascus_leucogenys	Pongo_abelii	Gorilla_gorilla	Pan_paniscus	Pan_troglodytes	Homo_sapiens
Echinops_telfairi	ns	ns	ns	ns	ns	ns	ns	ns	ns	ns	ns	ns	ns	ns	ns	ns	ns	ns	ns	ns	ns	ns	ns	ns	ns	ns	ns	ns	ns	ns	ns	ns	ns	ns	ns	ns	ns	*	ns	ns
Loxodonta_africana		ns	ns	ns	ns	ns	ns	ns	ns	ns	ns	ns	ns	ns	ns	ns	ns	ns	ns	ns	ns	ns	ns	ns	ns	ns	ns	ns	ns	ns	ns	ns	ns	ns	ns	ns	ns	ns	ns	ns
Trichechus_manatus_latirostris			ns	ns	ns	ns	ns	ns	ns	ns	ns	ns	ns	ns	ns	ns	ns	ns	ns	ns	ns	ns	ns	ns	ns	ns	ns	ns	ns	ns	ns	ns	ns	ns	ns	ns	ns	*	ns	ns
Sorex_araneus				ns	ns	ns	ns	ns	ns	ns	ns	ns	ns	ns	ns	ns	ns	ns	ns	ns	ns	ns	ns	ns	ns	ns	ns	ns	ns	ns	ns	ns	ns	ns	ns	ns	ns	*	ns	ns
Condylura_cristata					ns	ns	ns	ns	ns	ns	ns	ns	ns	ns	ns	ns	ns	ns	ns	ns	ns	ns	ns	ns	ns	ns	ns	ns	ns	ns	ns	ns	ns	ns	ns	ns	ns	ns	ns	ns
Equus_caballus						ns	ns	ns	ns	ns	ns	ns	ns	ns	ns	ns	ns	ns	ns	ns	ns	ns	ns	ns	ns	ns	ns	ns	ns	ns	ns	ns	ns	ns	ns	ns	ns	ns	ns	ns
Ceratotherium_simum_simum							ns	ns	ns	ns	*	ns	ns	ns	ns	ns	ns	ns	ns	ns	ns	ns	ns	ns	ns	ns	ns	ns	ns	ns	ns	ns	ns	ns	ns	ns	ns	**	ns	ns
Sus_scrofa								ns	ns	ns	ns	ns	ns	ns	ns	ns	ns	ns	ns	ns	ns	ns	ns	ns	ns	ns	ns	ns	ns	ns	ns	ns	ns	ns	ns	ns	ns	ns	ns	ns
Bos_taurus									ns	ns	ns	ns	ns	ns	ns	ns	ns	ns	ns	ns	ns	ns	ns	ns	ns	ns	ns	ns	ns	ns	ns	ns	ns	ns	ns	ns	ns	ns	ns	ns
Ovis_aries										ns	ns	ns	ns	ns	ns	ns	ns	ns	ns	ns	ns	ns	ns	ns	ns	ns	ns	ns	ns	ns	ns	ns	ns	ns	ns	ns	ns	ns	ns	ns
Orcinus_orca											ns	ns	ns	ns	ns	ns	ns	ns	ns	ns	ns	ns	ns	ns	ns	ns	ns	ns	ns	ns	ns	ns	ns	ns	ns	ns	ns	ns	ns	ns
Tursiops_truncatus												ns	ns	ns	*	ns	ns	ns	ns	*	ns	ns	ns	ns	*	ns	ns	ns	ns	ns	ns	ns	ns	ns	ns	ns	ns	ns	ns	ns
Felis_catus													ns	ns	ns	ns	ns	ns	ns	ns	ns	ns	ns	ns	ns	ns	ns	ns	ns	ns	ns	ns	ns	ns	ns	ns	ns	*	ns	ns
Ailuropoda_melanoleuca														ns	ns	ns	ns	ns	ns	ns	ns	ns	ns	ns	ns	ns	ns	ns	ns	ns	ns	ns	ns	ns	ns	ns	ns	*	ns	ns
Mustela_putorius_furo															ns	ns	ns	ns	ns	ns	ns	ns	ns	ns	ns	ns	ns	ns	ns	ns	ns	ns	ns	ns	ns	ns	ns	*	ns	ns
Odobenus_rosmarus_divergens																ns	ns	ns	ns	ns	ns	ns	ns	ns	ns	ns	ns	ns	ns	ns	ns	ns	ns	ns	ns	ns	ns	**	ns	ns
Canis_lupus_familiaris																	ns	ns	ns	ns	ns	ns	ns	ns	ns	ns	ns	ns	ns	ns	ns	ns	ns	ns	ns	ns	ns	*	ns	ns
Heterocephalus_glaber																		ns	ns	ns	ns	ns	ns	ns	ns	ns	ns	ns	ns	ns	ns	ns	ns	ns	ns	ns	ns	*	ns	ns
Octodon_degus																			ns	ns	ns	ns	ns	ns	ns	ns	ns	ns	ns	ns	ns	ns	ns	ns	ns	ns	ns	ns	ns	ns
Cavia_porcellus																				ns	ns	ns	ns	ns	ns	ns	ns	ns	ns	ns	ns	ns	ns	ns	ns	ns	ns	ns	ns	ns
Chinchilla_lanigera																					ns	ns	ns	ns	ns	ns	ns	ns	ns	ns	ns	ns	ns	ns	ns	ns	ns	**	ns	ns
Ictidomys_tridecemlineatus																						ns	ns	ns	ns	ns	ns	ns	ns	ns	ns	ns	ns	ns	ns	ns	ns	**	ns	ns
Jaculus_jaculus																							ns	ns	ns	ns	ns	ns	ns	ns	ns	ns	ns	ns	ns	ns	ns	**	ns	ns
Mus_musculus																								ns	ns	ns	ns	ns	ns	ns	ns	ns	ns	ns	ns	ns	ns	**	ns	ns
Rattus_norvegicus																									ns	ns	ns	ns	ns	ns	ns	ns	ns	ns	ns	ns	ns	**	ns	ns
Microtus_ochrogaster																										ns	ns	ns	ns	ns	ns	ns	ns	ns	ns	ns	ns	**	ns	ns
Cricetulus_griseus																											ns	ns	ns	ns	ns	ns	ns	ns	ns	ns	ns	ns	ns	ns
Mesocricetus_auratus																												ns	ns	ns	ns	ns	ns	ns	ns	ns	ns	*	ns	ns
Ochotona_princeps																													ns	ns	ns	ns	ns	ns	ns	ns	ns	*	ns	ns
Oryctolagus_cuniculus																														ns	ns	ns	ns	ns	ns	ns	ns	**	ns	ns
Otolemur_garnettii																															ns	ns	ns	ns	ns	ns	ns	**	ns	ns
Callithrix_jacchus																																ns	ns	ns	ns	ns	ns	ns	ns	ns
Saimiri_boliviensis																																	ns	ns	ns	ns	ns	ns	ns	ns
Macaca_mulatta																																		ns	ns	ns	ns	ns	ns	ns
Papio_anubis																																			ns	ns	ns	ns	ns	ns
Nomascus_leucogenys																																				ns	ns	ns	ns	ns
Pongo_abelii																																					ns	ns	ns	ns
Gorilla_gorilla																																						ns	ns	ns
Pan_paniscus																																							ns	ns
Pan_troglodytes																																								ns

Figure S2B. SFARI - Non-syndromic

	Loxodonta_africana	Trichechus_manatus_latirostris	Sorex_araneus	Condylura_cristata	Equus_caballus	Ceratotherium_simum_simum	Sus_scrofa	Bos_taurus	Ovis_aries	Orcinus_orca	Tursiops_truncatus	Felis_catus	Ailuropoda_melanoleuca	Mustela_putorius_furo	Odobenus_rosmarus_divergens	Canis_lupus_familiaris	Heterocephalus_glaber	Octodon_degus	Cavia_porcellus	Chinchilla_lanigera	Ictidomys_tridecemlineatus	Jaculus_jaculus	Mus_musculus	Rattus_norvegicus	Microtus_ochrogaster	Cricetulus_griseus	Mesocricetus_auratus	Ochotona_princeps	Oryctolagus_cuniculus	Otolemur_garnettii	Callithrix_jacchus	Saimiri_boliviensis	Macaca_mulatta	Papio_anubis	Nomascus_leucogenys	Pongo_abelii	Gorilla_gorilla	Pan_paniscus	Pan_troglodytes	Homo_sapiens
Echinops_telfairi	ns	ns	ns	ns	ns	ns	ns	ns	ns	*	****	ns	ns	ns	ns	ns	ns	ns	ns	ns	ns	ns	ns	ns	ns	ns	ns	ns	ns	ns	ns	ns	****	ns	ns	*	****	ns	****	ns
Loxodonta_africana		ns	ns	ns	ns	ns	ns	ns	ns	ns	****	ns	ns	ns	ns	ns	ns	ns	ns	ns	ns	ns	ns	ns	ns	ns	ns	ns	ns	ns	ns	ns	**	ns	ns	ns	**	ns	ns	ns
Trichechus_manatus_latirostris			ns	ns	ns	ns	ns	ns	ns	ns	****	ns	ns	ns	ns	ns	ns	ns	ns	ns	ns	ns	ns	ns	ns	ns	ns	ns	ns	ns	ns	ns	****	ns	ns	ns	****	ns	*	ns
Sorex_araneus				ns	ns	ns	ns	ns	ns	ns	****	ns	ns	ns	ns	ns	ns	ns	ns	ns	ns	ns	ns	ns	ns	ns	ns	ns	ns	ns	ns	ns	****	ns	ns	ns	****	ns	***	ns
Condylura_cristata					ns	ns	ns	ns	ns	*	****	ns	ns	ns	ns	ns	ns	ns	ns	ns	ns	ns	ns	ns	ns	ns	ns	ns	ns	ns	ns	ns	****	ns	ns	*	****	ns	****	*
Equus_caballus						ns	ns	ns	ns	ns	****	ns	ns	ns	ns	ns	ns	ns	ns	ns	ns	ns	ns	ns	ns	ns	ns	ns	ns	ns	ns	ns	***	ns	ns	ns	***	ns	ns	ns
Ceratotherium_simum_simum							ns	ns	ns	ns	****	ns	ns	ns	ns	ns	ns	ns	ns	ns	ns	ns	ns	ns	ns	ns	ns	ns	ns	ns	ns	ns	****	ns	ns	ns	****	ns	***	ns
Sus_scrofa								ns	ns	ns	****	ns	ns	ns	ns	ns	ns	ns	ns	ns	ns	ns	ns	ns	ns	ns	ns	ns	ns	ns	ns	ns	***	ns	ns	ns	***	ns	ns	ns
Bos_taurus									ns	ns	****	ns	ns	ns	ns	ns	ns	ns	ns	ns	ns	ns	ns	ns	ns	ns	ns	ns	ns	ns	ns	ns	****	ns	ns	ns	****	ns	**	ns
Ovis_aries										ns	****	ns	ns	ns	ns	ns	ns	ns	ns	ns	ns	ns	ns	ns	ns	ns	ns	ns	ns	ns	ns	ns	ns	ns	ns	ns	ns	ns	ns	ns
Orcinus_orca											****	ns	ns	*	*	ns	ns	*	ns	*	ns	*	**	**	**	ns	ns	**	ns	ns	ns	ns	ns	ns	ns	ns	ns	ns	ns	ns
Tursiops_truncatus												****	****	****	****	****	****	****	****	****	****	****	****	****	****	****	****	****	****	****	****	****	**	****	****	****	**	****	****	****
Felis_catus													ns	ns	ns	ns	ns	ns	ns	ns	ns	ns	ns	ns	ns	ns	ns	ns	ns	ns	ns	ns	***	ns	ns	ns	***	ns	ns	ns
Ailuropoda_melanoleuca														ns	ns	ns	ns	ns	ns	ns	ns	ns	ns	ns	ns	ns	ns	ns	ns	ns	ns	ns	***	ns	ns	ns	***	ns	ns	ns
Mustela_putorius_furo															ns	ns	ns	ns	ns	ns	ns	ns	ns	ns	ns	ns	ns	ns	ns	ns	ns	ns	****	ns	ns	*	****	ns	****	*
Odobenus_rosmarus_divergens																ns	ns	ns	ns	ns	ns	ns	ns	ns	ns	ns	ns	ns	ns	ns	ns	ns	****	ns	ns	*	****	ns	****	ns
Canis_lupus_familiaris																	ns	ns	ns	ns	ns	ns	ns	ns	ns	ns	ns	ns	ns	ns	ns	ns	****	ns	ns	ns	****	ns	****	ns
Heterocephalus_glaber																		ns	ns	ns	ns	ns	ns	ns	ns	ns	ns	ns	ns	ns	ns	ns	****	ns	ns	ns	****	ns	***	ns
Octodon_degus																			ns	ns	ns	ns	ns	ns	ns	ns	ns	ns	ns	ns	ns	ns	****	ns	ns	*	****	ns	****	*
Cavia_porcellus																				ns	ns	ns	ns	ns	ns	ns	ns	ns	ns	ns	ns	ns	****	ns	ns	*	****	ns	****	ns
Chinchilla_lanigera																					ns	ns	ns	ns	ns	ns	ns	ns	ns	ns	ns	ns	****	ns	ns	*	****	ns	****	ns
Ictidomys_tridecemlineatus																						ns	ns	ns	ns	ns	ns	ns	ns	ns	ns	ns	****	ns	ns	ns	****	ns	***	ns
Jaculus_jaculus																							ns	ns	ns	ns	ns	ns	ns	ns	ns	ns	****	ns	ns	*	****	ns	****	*
Mus_musculus																								ns	ns	ns	ns	ns	ns	ns	ns	ns	****	*	*	***	****	ns	****	**
Rattus_norvegicus																									ns	ns	ns	ns	ns	ns	ns	ns	****	*	*	***	****	ns	****	**
Microtus_ochrogaster																										ns	ns	ns	ns	ns	ns	ns	****	*	*	**	****	ns	****	**
Cricetulus_griseus																											ns	ns	ns	ns	ns	ns	*	ns	ns	ns	*	ns	ns	ns
Mesocricetus_auratus																												ns	ns	ns	ns	ns	***	ns	ns	ns	***	ns	ns	ns
Ochotona_princeps																													ns	ns	ns	ns	****	*	*	**	****	ns	****	**
Oryctolagus_cuniculus																														ns	ns	ns	****	ns	ns	ns	****	ns	**	ns
Otolemur_garnettii																															ns	ns	****	ns	ns	ns	****	ns	***	ns
Callithrix_jacchus																																ns	*	ns	ns	ns	**	ns	ns	ns
Saimiri_boliviensis																																	**	ns	ns	ns	***	ns	ns	ns
Macaca_mulatta																																		ns	ns	ns	ns	ns	ns	ns
Papio_anubis																																			ns	ns	ns	ns	ns	ns
Nomascus_leucogenys																																				ns	ns	ns	ns	ns
Pongo_abelii																																					ns	ns	ns	ns
Gorilla_gorilla																																						ns	ns	ns
Pan_paniscus																																							ns	ns
Pan_troglodytes																																								ns

Figure S3A. SFARI - All scored genes

	Loxodonta_africana	Trichechus_manatus_latirostris	Sorex_araneus	Condylura_cristata	Equus_caballus	Ceratotherium_simum_simum	Sus_scrofa	Bos_taurus	Ovis_aries	Orcinus_orca	Tursiops_truncatus	Felis_catus	Ailuropoda_melanoleuca	Mustela_putorius_furo	Odobenus_rosmarus_divergens	Canis_lupus_familiaris	Heterocephalus_glaber	Octodon_degus	Cavia_porcellus	Chinchilla_lanigera	Ictidomys_tridecemlineatus	Jaculus_jaculus	Mus_musculus	Rattus_norvegicus	Microtus_ochrogaster	Cricetulus_griseus	Mesocricetus_auratus	Ochotona_princeps	Oryctolagus_cuniculus	Otolemur_garnettii	Callithrix_jacchus	Saimiri_boliviensis	Macaca_mulatta	Papio_anubis	Nomascus_leucogenys	Pongo_abelii	Gorilla_gorilla	Pan_paniscus	Pan_troglodytes	Homo_sapiens
Echinops_telfairi	ns	ns	ns	ns	ns	ns	ns	ns	ns	*	****	ns	ns	ns	ns	ns	ns	ns	ns	ns	ns	ns	ns	ns	ns	ns	ns	ns	ns	ns	ns	ns	****	*	ns	**	****	*	****	*
Loxodonta_africana		ns	ns	ns	ns	ns	ns	ns	ns	ns	****	ns	ns	ns	ns	ns	ns	ns	ns	ns	ns	ns	ns	ns	ns	ns	ns	ns	ns	ns	ns	ns	***	ns	ns	ns	**	ns	ns	ns
Trichechus_manatus_latirostris			ns	ns	ns	ns	ns	ns	ns	ns	****	ns	ns	ns	ns	ns	ns	ns	ns	ns	ns	ns	ns	ns	ns	ns	ns	ns	ns	ns	ns	ns	****	ns	ns	ns	****	ns	***	ns
Sorex_araneus				ns	ns	ns	ns	ns	ns	ns	****	ns	ns	ns	ns	ns	ns	ns	ns	ns	ns	ns	ns	ns	ns	ns	ns	ns	ns	ns	ns	ns	****	ns	ns	*	****	ns	****	ns
Condylura_cristata					ns	ns	ns	ns	ns	*	****	ns	ns	ns	ns	ns	ns	ns	ns	ns	ns	ns	ns	ns	ns	ns	ns	ns	ns	ns	ns	ns	****	*	ns	**	****	**	****	*
Equus_caballus						ns	ns	ns	ns	ns	****	ns	ns	ns	ns	ns	ns	ns	ns	ns	ns	ns	ns	ns	ns	ns	ns	ns	ns	ns	ns	ns	****	ns	ns	ns	****	ns	**	ns
Ceratotherium_simum_simum							ns	ns	ns	ns	****	ns	ns	ns	ns	ns	ns	ns	ns	ns	ns	ns	ns	ns	ns	ns	ns	ns	ns	ns	ns	ns	****	ns	ns	**	****	*	****	*
Sus_scrofa								ns	ns	ns	****	ns	ns	ns	ns	ns	ns	ns	ns	ns	ns	ns	ns	ns	ns	ns	ns	ns	ns	ns	ns	ns	****	ns	ns	ns	****	ns	*	ns
Bos_taurus									ns	ns	****	ns	ns	ns	ns	ns	ns	ns	ns	ns	ns	ns	ns	ns	ns	ns	ns	ns	ns	ns	ns	ns	****	ns	ns	ns	****	ns	***	ns
Ovis_aries										ns	****	ns	ns	ns	ns	ns	ns	ns	ns	ns	ns	ns	ns	ns	ns	ns	ns	ns	ns	ns	ns	ns	*	ns	ns	ns	*	ns	ns	ns
Orcinus_orca											****	ns	ns	*	*	ns	ns	*	ns	*	ns	*	**	**	**	ns	ns	**	ns	ns	ns	ns	ns	ns	ns	ns	ns	ns	ns	ns
Tursiops_truncatus												****	****	****	****	****	****	****	****	****	****	****	****	****	****	****	****	****	****	****	****	****	**	****	****	****	**	****	****	****
Felis_catus													ns	ns	ns	ns	ns	ns	ns	ns	ns	ns	ns	ns	ns	ns	ns	ns	ns	ns	ns	ns	****	ns	ns	ns	****	ns	**	ns
Ailuropoda_melanoleuca														ns	ns	ns	ns	ns	ns	ns	ns	ns	ns	ns	ns	ns	ns	ns	ns	ns	ns	ns	****	ns	ns	ns	****	ns	*	ns
Mustela_putorius_furo															ns	ns	ns	ns	ns	ns	ns	ns	ns	ns	ns	ns	ns	ns	ns	ns	ns	ns	****	*	ns	***	****	**	****	*
Odobenus_rosmarus_divergens																ns	ns	ns	ns	ns	ns	ns	ns	ns	ns	ns	ns	ns	ns	ns	ns	ns	****	*	ns	***	****	**	****	*
Canis_lupus_familiaris																	ns	ns	ns	ns	ns	ns	ns	ns	ns	ns	ns	ns	ns	ns	ns	ns	****	ns	ns	**	****	*	****	*
Heterocephalus_glaber																		ns	ns	ns	ns	ns	ns	ns	ns	ns	ns	ns	ns	ns	ns	ns	****	ns	ns	**	****	ns	****	ns
Octodon_degus																			ns	ns	ns	ns	ns	ns	ns	ns	ns	ns	ns	ns	ns	ns	****	*	ns	***	****	**	****	*
Cavia_porcellus																				ns	ns	ns	ns	ns	ns	ns	ns	ns	ns	ns	ns	ns	****	ns	ns	**	****	*	****	ns
Chinchilla_lanigera																					ns	ns	ns	ns	ns	ns	ns	ns	ns	ns	ns	ns	****	*	ns	***	****	**	****	**
Ictidomys_tridecemlineatus																						ns	ns	ns	ns	ns	ns	ns	ns	ns	ns	ns	****	ns	ns	**	****	*	****	*
Jaculus_jaculus																							ns	ns	ns	ns	ns	ns	ns	ns	ns	ns	****	**	*	***	****	**	****	**
Mus_musculus																								ns	ns	ns	ns	ns	ns	ns	ns	ns	****	***	**	****	****	***	****	***
Rattus_norvegicus																									ns	ns	ns	ns	ns	ns	ns	ns	****	***	**	****	****	***	****	***
Microtus_ochrogaster																										ns	ns	ns	ns	ns	ns	ns	****	***	**	****	****	***	****	***
Cricetulus_griseus																											ns	ns	ns	ns	ns	ns	**	ns	ns	ns	*	ns	ns	ns
Mesocricetus_auratus																												ns	ns	ns	ns	ns	****	ns	ns	ns	****	ns	*	ns
Ochotona_princeps																													ns	ns	ns	ns	****	**	*	****	****	***	****	**
Oryctolagus_cuniculus																														ns	ns	ns	****	ns	ns	ns	****	ns	***	ns
Otolemur_garnettii																															ns	ns	****	ns	ns	**	****	*	****	ns
Callithrix_jacchus																																ns	**	ns	ns	ns	**	ns	ns	ns
Saimiri_boliviensis																																	***	ns	ns	ns	***	ns	ns	ns
Macaca_mulatta																																		ns	ns	ns	ns	ns	ns	ns
Papio_anubis																																			ns	ns	ns	ns	ns	ns
Nomascus_leucogenys																																				ns	ns	ns	ns	ns
Pongo_abelii																																					ns	ns	ns	ns
Gorilla_gorilla																																						ns	ns	ns
Pan_paniscus																																							ns	ns
Pan_troglodytes																																								ns

Figure S3B. SFARI - Genes scored 5+ (hypothesized+)

	Loxodonta_africana	Trichechus_manatus_latirostris	Sorex_araneus	Condylura_cristata	Equus_caballus	Ceratotherium_simum_simum	Sus_scrofa	Bos_taurus	Ovis_aries	Orcinus_orca	Tursiops_truncatus	Felis_catus	Ailuropoda_melanoleuca	Mustela_putorius_furo	Odobenus_rosmarus_divergens	Canis_lupus_familiaris	Heterocephalus_glaber	Octodon_degus	Cavia_porcellus	Chinchilla_lanigera	Ictidomys_tridecemlineatus	Jaculus_jaculus	Mus_musculus	Rattus_norvegicus	Microtus_ochrogaster	Cricetulus_griseus	Mesocricetus_auratus	Ochotona_princeps	Oryctolagus_cuniculus	Otolemur_garnettii	Callithrix_jacchus	Saimiri_boliviensis	Macaca_mulatta	Papio_anubis	Nomascus_leucogenys	Pongo_abelii	Gorilla_gorilla	Pan_paniscus	Pan_troglodytes	Homo_sapiens
Echinops_telfairi	ns	ns	ns	ns	ns	ns	ns	ns	ns	*	****	ns	ns	ns	ns	ns	ns	ns	ns	ns	ns	ns	ns	ns	ns	ns	ns	ns	ns	ns	ns	ns	****	ns	ns	ns	****	ns	****	*
Loxodonta_africana		ns	ns	ns	ns	ns	ns	ns	ns	ns	****	ns	ns	ns	ns	ns	ns	ns	ns	ns	ns	ns	ns	ns	ns	ns	ns	ns	ns	ns	ns	ns	ns	ns	ns	ns	ns	ns	ns	ns
Trichechus_manatus_latirostris			ns	ns	ns	ns	ns	ns	ns	ns	****	ns	ns	ns	ns	ns	ns	ns	ns	ns	ns	ns	ns	ns	ns	ns	ns	ns	ns	ns	ns	ns	**	ns	ns	ns	***	ns	**	ns
Sorex_araneus				ns	ns	ns	ns	ns	ns	ns	****	ns	ns	ns	ns	ns	ns	ns	ns	ns	ns	ns	ns	ns	ns	ns	ns	ns	ns	ns	ns	ns	***	ns	ns	ns	****	ns	***	ns
Condylura_cristata					ns	ns	ns	ns	ns	**	****	ns	ns	ns	ns	ns	ns	ns	ns	ns	ns	ns	ns	ns	ns	ns	ns	ns	ns	ns	ns	ns	****	ns	ns	ns	****	ns	****	*
Equus_caballus						ns	ns	ns	ns	ns	****	ns	ns	ns	ns	ns	ns	ns	ns	ns	ns	ns	ns	ns	ns	ns	ns	ns	ns	ns	ns	ns	*	ns	ns	ns	**	ns	ns	ns
Ceratotherium_simum_simum							ns	ns	ns	*	****	ns	ns	ns	ns	ns	ns	ns	ns	ns	ns	ns	ns	ns	ns	ns	ns	ns	ns	ns	ns	ns	****	ns	ns	ns	****	ns	***	ns
Sus_scrofa								ns	ns	ns	****	ns	ns	ns	ns	ns	ns	ns	ns	ns	ns	ns	ns	ns	ns	ns	ns	ns	ns	ns	ns	ns	*	ns	ns	ns	**	ns	ns	ns
Bos_taurus									ns	ns	****	ns	ns	ns	ns	ns	ns	ns	ns	ns	ns	ns	ns	ns	ns	ns	ns	ns	ns	ns	ns	ns	***	ns	ns	ns	****	ns	***	ns
Ovis_aries										ns	****	ns	ns	ns	ns	ns	ns	ns	ns	ns	ns	ns	ns	ns	ns	ns	ns	ns	ns	ns	ns	ns	ns	ns	ns	ns	ns	ns	ns	ns
Orcinus_orca											****	ns	ns	**	*	*	ns	**	*	**	*	**	***	***	***	ns	ns	**	ns	ns	ns	ns	ns	ns	ns	ns	ns	ns	ns	ns
Tursiops_truncatus												****	****	****	****	****	****	****	****	****	****	****	****	****	****	****	****	****	****	****	****	****	***	****	****	****	**	****	***	****
Felis_catus													ns	ns	ns	ns	ns	ns	ns	ns	ns	ns	ns	ns	ns	ns	ns	ns	ns	ns	ns	ns	ns	ns	ns	ns	**	ns	ns	ns
Ailuropoda_melanoleuca														ns	ns	ns	ns	ns	ns	ns	ns	ns	ns	ns	ns	ns	ns	ns	ns	ns	ns	ns	*	ns	ns	ns	**	ns	*	ns
Mustela_putorius_furo															ns	ns	ns	ns	ns	ns	ns	ns	ns	ns	ns	ns	ns	ns	ns	ns	ns	ns	****	ns	ns	ns	****	ns	****	*
Odobenus_rosmarus_divergens																ns	ns	ns	ns	ns	ns	ns	ns	ns	ns	ns	ns	ns	ns	ns	ns	ns	****	ns	ns	ns	****	ns	****	ns
Canis_lupus_familiaris																	ns	ns	ns	ns	ns	ns	ns	ns	ns	ns	ns	ns	ns	ns	ns	ns	****	ns	ns	ns	****	ns	****	ns
Heterocephalus_glaber																		ns	ns	ns	ns	ns	ns	ns	ns	ns	ns	ns	ns	ns	ns	ns	***	ns	ns	ns	****	ns	***	ns
Octodon_degus																			ns	ns	ns	ns	ns	ns	ns	ns	ns	ns	ns	ns	ns	ns	****	ns	ns	ns	****	ns	****	*
Cavia_porcellus																				ns	ns	ns	ns	ns	ns	ns	ns	ns	ns	ns	ns	ns	****	ns	ns	ns	****	ns	****	ns
Chinchilla_lanigera																					ns	ns	ns	ns	ns	ns	ns	ns	ns	ns	ns	ns	****	ns	ns	ns	****	ns	****	*
Ictidomys_tridecemlineatus																						ns	ns	ns	ns	ns	ns	ns	ns	ns	ns	ns	****	ns	ns	ns	****	ns	****	ns
Jaculus_jaculus																							ns	ns	ns	ns	ns	ns	ns	ns	ns	ns	****	ns	ns	ns	****	ns	****	*
Mus_musculus																								ns	ns	ns	ns	ns	ns	ns	ns	ns	****	ns	ns	*	****	ns	****	**
Rattus_norvegicus																									ns	ns	ns	ns	ns	ns	ns	ns	****	ns	ns	*	****	ns	****	**
Microtus_ochrogaster																										ns	ns	ns	ns	ns	ns	ns	****	ns	ns	ns	****	ns	****	**
Cricetulus_griseus																											ns	ns	ns	ns	ns	ns	ns	ns	ns	ns	ns	ns	ns	ns
Mesocricetus_auratus																												ns	ns	ns	ns	ns	ns	ns	ns	ns	**	ns	ns	ns
Ochotona_princeps																													ns	ns	ns	ns	****	ns	ns	ns	****	ns	****	**
Oryctolagus_cuniculus																														ns	ns	ns	**	ns	ns	ns	***	ns	**	ns
Otolemur_garnettii																															ns	ns	****	ns	ns	ns	****	ns	***	ns
Callithrix_jacchus																																ns	ns	ns	ns	ns	*	ns	ns	ns
Saimiri_boliviensis																																	ns	ns	ns	ns	*	ns	ns	ns
Macaca_mulatta																																		ns	ns	ns	ns	ns	ns	ns
Papio_anubis																																			ns	ns	ns	ns	ns	ns
Nomascus_leucogenys																																				ns	ns	ns	ns	ns
Pongo_abelii																																					ns	ns	ns	ns
Gorilla_gorilla																																						ns	ns	ns
Pan_paniscus																																							ns	ns
Pan_troglodytes																																								ns

Figure S3C. SFARI - Genes scored 4+ (minimal evidence+)

	Loxodonta_africana	Trichechus_manatus_latirostris	Sorex_araneus	Condylura_cristata	Equus_caballus	Ceratotherium_simum_simum	Sus_scrofa	Bos_taurus	Ovis_aries	Orcinus_orca	Tursiops_truncatus	Felis_catus	Ailuropoda_melanoleuca	Mustela_putorius_furo	Odobenus_rosmarus_divergens	Canis_lupus_familiaris	Heterocephalus_glaber	Octodon_degus	Cavia_porcellus	Chinchilla_lanigera	Ictidomys_tridecemlineatus	Jaculus_jaculus	Mus_musculus	Rattus_norvegicus	Microtus_ochrogaster	Cricetulus_griseus	Mesocricetus_auratus	Ochotona_princeps	Oryctolagus_cuniculus	Otolemur_garnettii	Callithrix_jacchus	Saimiri_boliviensis	Macaca_mulatta	Papio_anubis	Nomascus_leucogenys	Pongo_abelii	Gorilla_gorilla	Pan_paniscus	Pan_troglodytes	Homo_sapiens
Echinops_telfairi	ns	ns	ns	ns	ns	ns	ns	ns	ns	ns	****	ns	ns	ns	ns	ns	ns	ns	ns	ns	ns	ns	ns	ns	ns	ns	ns	ns	ns	ns	ns	ns	***	ns	ns	ns	***	ns	***	ns
Loxodonta_africana		ns	ns	ns	ns	ns	ns	ns	ns	ns	****	ns	ns	ns	ns	ns	ns	ns	ns	ns	ns	ns	ns	ns	ns	ns	ns	ns	ns	ns	ns	ns	ns	ns	ns	ns	ns	ns	ns	ns
Trichechus_manatus_latirostris			ns	ns	ns	ns	ns	ns	ns	ns	****	ns	ns	ns	ns	ns	ns	ns	ns	ns	ns	ns	ns	ns	ns	ns	ns	ns	ns	ns	ns	ns	**	ns	ns	ns	**	ns	*	ns
Sorex_araneus				ns	ns	ns	ns	ns	ns	ns	****	ns	ns	ns	ns	ns	ns	ns	ns	ns	ns	ns	ns	ns	ns	ns	ns	ns	ns	ns	ns	ns	*	ns	ns	ns	**	ns	*	ns
Condylura_cristata					ns	ns	ns	ns	ns	ns	****	ns	ns	ns	ns	ns	ns	ns	ns	ns	ns	ns	ns	ns	ns	ns	ns	ns	ns	ns	ns	ns	****	ns	ns	ns	****	ns	***	ns
Equus_caballus						ns	ns	ns	ns	ns	****	ns	ns	ns	ns	ns	ns	ns	ns	ns	ns	ns	ns	ns	ns	ns	ns	ns	ns	ns	ns	ns	ns	ns	ns	ns	ns	ns	ns	ns
Ceratotherium_simum_simum							ns	ns	ns	ns	****	ns	ns	ns	ns	ns	ns	ns	ns	ns	ns	ns	ns	ns	ns	ns	ns	ns	ns	ns	ns	ns	***	ns	ns	ns	***	ns	**	ns
Sus_scrofa								ns	ns	ns	****	ns	ns	ns	ns	ns	ns	ns	ns	ns	ns	ns	ns	ns	ns	ns	ns	ns	ns	ns	ns	ns	ns	ns	ns	ns	*	ns	ns	ns
Bos_taurus									ns	ns	****	ns	ns	ns	ns	ns	ns	ns	ns	ns	ns	ns	ns	ns	ns	ns	ns	ns	ns	ns	ns	ns	**	ns	ns	ns	**	ns	**	ns
Ovis_aries										ns	****	ns	ns	ns	ns	ns	ns	ns	ns	ns	ns	ns	ns	ns	ns	ns	ns	ns	ns	ns	ns	ns	ns	ns	ns	ns	ns	ns	ns	ns
Orcinus_orca											****	ns	ns	ns	ns	ns	ns	ns	ns	ns	ns	ns	ns	ns	ns	ns	ns	ns	ns	ns	ns	ns	ns	ns	ns	ns	ns	ns	ns	ns
Tursiops_truncatus												****	****	****	****	****	****	****	****	****	****	****	****	****	****	****	****	****	****	****	****	****	*	****	****	****	ns	****	*	****
Felis_catus													ns	ns	ns	ns	ns	ns	ns	ns	ns	ns	ns	ns	ns	ns	ns	ns	ns	ns	ns	ns	ns	ns	ns	ns	ns	ns	ns	ns
Ailuropoda_melanoleuca														ns	ns	ns	ns	ns	ns	ns	ns	ns	ns	ns	ns	ns	ns	ns	ns	ns	ns	ns	ns	ns	ns	ns	*	ns	ns	ns
Mustela_putorius_furo															ns	ns	ns	ns	ns	ns	ns	ns	ns	ns	ns	ns	ns	ns	ns	ns	ns	ns	***	ns	ns	ns	****	ns	***	ns
Odobenus_rosmarus_divergens																ns	ns	ns	ns	ns	ns	ns	ns	ns	ns	ns	ns	ns	ns	ns	ns	ns	***	ns	ns	ns	***	ns	**	ns
Canis_lupus_familiaris																	ns	ns	ns	ns	ns	ns	ns	ns	ns	ns	ns	ns	ns	ns	ns	ns	****	ns	ns	ns	****	ns	***	ns
Heterocephalus_glaber																		ns	ns	ns	ns	ns	ns	ns	ns	ns	ns	ns	ns	ns	ns	ns	***	ns	ns	ns	***	ns	**	ns
Octodon_degus																			ns	ns	ns	ns	ns	ns	ns	ns	ns	ns	ns	ns	ns	ns	****	ns	ns	ns	****	ns	***	ns
Cavia_porcellus																				ns	ns	ns	ns	ns	ns	ns	ns	ns	ns	ns	ns	ns	***	ns	ns	ns	***	ns	***	ns
Chinchilla_lanigera																					ns	ns	ns	ns	ns	ns	ns	ns	ns	ns	ns	ns	****	ns	ns	ns	****	ns	***	ns
Ictidomys_tridecemlineatus																						ns	ns	ns	ns	ns	ns	ns	ns	ns	ns	ns	***	ns	ns	ns	***	ns	**	ns
Jaculus_jaculus																							ns	ns	ns	ns	ns	ns	ns	ns	ns	ns	****	ns	ns	ns	****	ns	***	ns
Mus_musculus																								ns	ns	ns	ns	ns	ns	ns	ns	ns	****	ns	ns	ns	****	ns	****	ns
Rattus_norvegicus																									ns	ns	ns	ns	ns	ns	ns	ns	****	ns	ns	ns	****	ns	****	ns
Microtus_ochrogaster																										ns	ns	ns	ns	ns	ns	ns	****	ns	ns	ns	****	ns	****	ns
Cricetulus_griseus																											ns	ns	ns	ns	ns	ns	ns	ns	ns	ns	ns	ns	ns	ns
Mesocricetus_auratus																												ns	ns	ns	ns	ns	ns	ns	ns	ns	ns	ns	ns	ns
Ochotona_princeps																													ns	ns	ns	ns	****	ns	ns	ns	****	ns	****	ns
Oryctolagus_cuniculus																														ns	ns	ns	*	ns	ns	ns	*	ns	ns	ns
Otolemur_garnettii																															ns	ns	**	ns	ns	ns	***	ns	**	ns
Callithrix_jacchus																																ns	ns	ns	ns	ns	ns	ns	ns	ns
Saimiri_boliviensis																																	ns	ns	ns	ns	ns	ns	ns	ns
Macaca_mulatta																																		ns	ns	ns	ns	ns	ns	ns
Papio_anubis																																			ns	ns	ns	ns	ns	ns
Nomascus_leucogenys																																				ns	ns	ns	ns	ns
Pongo_abelii																																					ns	ns	ns	ns
Gorilla_gorilla																																						ns	ns	ns
Pan_paniscus																																							ns	ns
Pan_troglodytes																																								ns

Figure S3D. SFARI - Genes scored 3+ (suggestive evidence+)

	Loxodonta_africana	Trichechus_manatus_latirostris	Sorex_araneus	Condylura_cristata	Equus_caballus	Ceratotherium_simum_simum	Sus_scrofa	Bos_taurus	Ovis_aries	Orcinus_orca	Tursiops_truncatus	Felis_catus	Ailuropoda_melanoleuca	Mustela_putorius_furo	Odobenus_rosmarus_divergens	Canis_lupus_familiaris	Heterocephalus_glaber	Octodon_degus	Cavia_porcellus	Chinchilla_lanigera	Ictidomys_tridecemlineatus	Jaculus_jaculus	Mus_musculus	Rattus_norvegicus	Microtus_ochrogaster	Cricetulus_griseus	Mesocricetus_auratus	Ochotona_princeps	Oryctolagus_cuniculus	Otolemur_garnettii	Callithrix_jacchus	Saimiri_boliviensis	Macaca_mulatta	Papio_anubis	Nomascus_leucogenys	Pongo_abelii	Gorilla_gorilla	Pan_paniscus	Pan_troglodytes	Homo_sapiens
Echinops_telfairi	ns	ns	ns	ns	ns	ns	ns	ns	ns	ns	****	ns	ns	ns	ns	ns	ns	ns	ns	ns	ns	ns	ns	ns	ns	ns	ns	ns	ns	ns	ns	ns	ns	ns	ns	ns	ns	ns	ns	ns
Loxodonta_africana		ns	ns	ns	ns	ns	ns	ns	ns	ns	****	ns	ns	ns	ns	ns	ns	ns	ns	ns	ns	ns	ns	ns	ns	ns	ns	ns	ns	ns	ns	ns	ns	ns	ns	ns	ns	ns	ns	ns
Trichechus_manatus_latirostris			ns	ns	ns	ns	ns	ns	ns	ns	****	ns	ns	ns	ns	ns	ns	ns	ns	ns	ns	ns	ns	ns	ns	ns	ns	ns	ns	ns	ns	ns	ns	ns	ns	ns	ns	ns	ns	ns
Sorex_araneus				ns	ns	ns	ns	ns	ns	ns	***	ns	ns	ns	ns	ns	ns	ns	ns	ns	ns	ns	ns	ns	ns	ns	ns	ns	ns	ns	ns	ns	ns	ns	ns	ns	ns	ns	ns	ns
Condylura_cristata					ns	ns	ns	ns	ns	ns	****	ns	ns	ns	ns	ns	ns	ns	ns	ns	ns	ns	ns	ns	ns	ns	ns	ns	ns	ns	ns	ns	ns	ns	ns	ns	ns	ns	ns	ns
Equus_caballus						ns	ns	ns	ns	ns	****	ns	ns	ns	ns	ns	ns	ns	ns	ns	ns	ns	ns	ns	ns	ns	ns	ns	ns	ns	ns	ns	ns	ns	ns	ns	ns	ns	ns	ns
Ceratotherium_simum_simum							ns	ns	ns	ns	****	ns	ns	ns	ns	ns	ns	ns	ns	ns	ns	ns	ns	ns	ns	ns	ns	ns	ns	ns	ns	ns	ns	ns	ns	ns	ns	ns	ns	ns
Sus_scrofa								ns	ns	ns	***	ns	ns	ns	ns	ns	ns	ns	ns	ns	ns	ns	ns	ns	ns	ns	ns	ns	ns	ns	ns	ns	ns	ns	ns	ns	ns	ns	ns	ns
Bos_taurus									ns	ns	****	ns	ns	ns	ns	ns	ns	ns	ns	ns	ns	ns	ns	ns	ns	ns	ns	ns	ns	ns	ns	ns	ns	ns	ns	ns	ns	ns	ns	ns
Ovis_aries										ns	ns	ns	ns	ns	ns	ns	ns	ns	ns	ns	ns	ns	ns	ns	ns	ns	ns	ns	ns	ns	ns	ns	ns	ns	ns	ns	ns	ns	ns	ns
Orcinus_orca											****	ns	ns	ns	ns	ns	ns	ns	ns	ns	ns	ns	ns	ns	ns	ns	ns	ns	ns	ns	ns	ns	ns	ns	ns	ns	ns	ns	ns	ns
Tursiops_truncatus												****	****	****	****	****	****	****	****	****	****	****	****	****	****	****	**	****	****	****	***	***	*	*	*	**	ns	***	ns	****
Felis_catus													ns	ns	ns	ns	ns	ns	ns	ns	ns	ns	ns	ns	ns	ns	ns	ns	ns	ns	ns	ns	ns	ns	ns	ns	ns	ns	ns	ns
Ailuropoda_melanoleuca														ns	ns	ns	ns	ns	ns	ns	ns	ns	ns	ns	ns	ns	ns	ns	ns	ns	ns	ns	ns	ns	ns	ns	ns	ns	ns	ns
Mustela_putorius_furo															ns	ns	ns	ns	ns	ns	ns	ns	ns	ns	ns	ns	ns	ns	ns	ns	ns	ns	ns	ns	ns	ns	ns	ns	ns	ns
Odobenus_rosmarus_divergens																ns	ns	ns	ns	ns	ns	ns	ns	ns	ns	ns	ns	ns	ns	ns	ns	ns	ns	ns	ns	ns	ns	ns	ns	ns
Canis_lupus_familiaris																	ns	ns	ns	ns	ns	ns	ns	ns	ns	ns	ns	ns	ns	ns	ns	ns	ns	ns	ns	ns	ns	ns	ns	ns
Heterocephalus_glaber																		ns	ns	ns	ns	ns	ns	ns	ns	ns	ns	ns	ns	ns	ns	ns	ns	ns	ns	ns	ns	ns	ns	ns
Octodon_degus																			ns	ns	ns	ns	ns	ns	ns	ns	ns	ns	ns	ns	ns	ns	ns	ns	ns	ns	ns	ns	ns	ns
Cavia_porcellus																				ns	ns	ns	ns	ns	ns	ns	ns	ns	ns	ns	ns	ns	ns	ns	ns	ns	ns	ns	ns	ns
Chinchilla_lanigera																					ns	ns	ns	ns	ns	ns	ns	ns	ns	ns	ns	ns	ns	ns	ns	ns	ns	ns	ns	ns
Ictidomys_tridecemlineatus																						ns	ns	ns	ns	ns	ns	ns	ns	ns	ns	ns	ns	ns	ns	ns	ns	ns	ns	ns
Jaculus_jaculus																							ns	ns	ns	ns	ns	ns	ns	ns	ns	ns	ns	ns	ns	ns	ns	ns	ns	ns
Mus_musculus																								ns	ns	ns	ns	ns	ns	ns	ns	ns	ns	ns	ns	ns	ns	ns	ns	ns
Rattus_norvegicus																									ns	ns	ns	ns	ns	ns	ns	ns	ns	ns	ns	ns	ns	ns	ns	ns
Microtus_ochrogaster																										ns	ns	ns	ns	ns	ns	ns	ns	ns	ns	ns	ns	ns	ns	ns
Cricetulus_griseus																											ns	ns	ns	ns	ns	ns	ns	ns	ns	ns	ns	ns	ns	ns
Mesocricetus_auratus																												ns	ns	ns	ns	ns	ns	ns	ns	ns	ns	ns	ns	ns
Ochotona_princeps																													ns	ns	ns	ns	ns	ns	ns	ns	ns	ns	ns	ns
Oryctolagus_cuniculus																														ns	ns	ns	ns	ns	ns	ns	ns	ns	ns	ns
Otolemur_garnettii																															ns	ns	ns	ns	ns	ns	ns	ns	ns	ns
Callithrix_jacchus																																ns	ns	ns	ns	ns	ns	ns	ns	ns
Saimiri_boliviensis																																	ns	ns	ns	ns	ns	ns	ns	ns
Macaca_mulatta																																		ns	ns	ns	ns	ns	ns	ns
Papio_anubis																																			ns	ns	ns	ns	ns	ns
Nomascus_leucogenys																																				ns	ns	ns	ns	ns
Pongo_abelii																																					ns	ns	ns	ns
Gorilla_gorilla																																						ns	ns	ns
Pan_paniscus																																							ns	ns
Pan_troglodytes																																								ns
