# Supplementary figures and images for: Evolutionary conservation in genes underlying human psychiatric disorders
Source: Front Hum Neurosci. 2014 May 6;8:283. doi: 10.3389/fnhum.2014.00283 (PMC4018557; doi:10.3389/fnhum.2014.00283)

SzGR Ng et al.

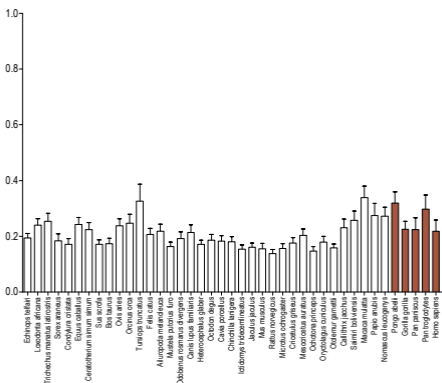

SzGR Lewis et al.

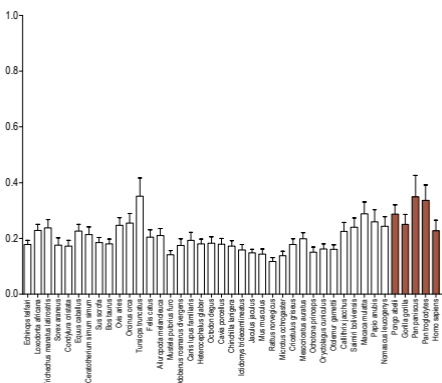

SzGR COR

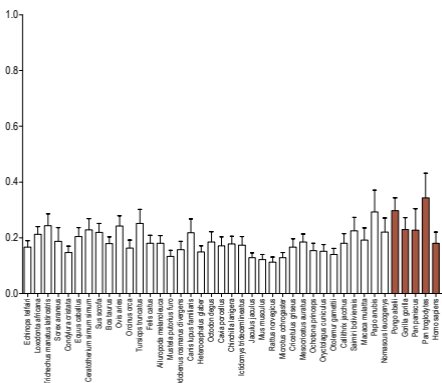

SzGR Core

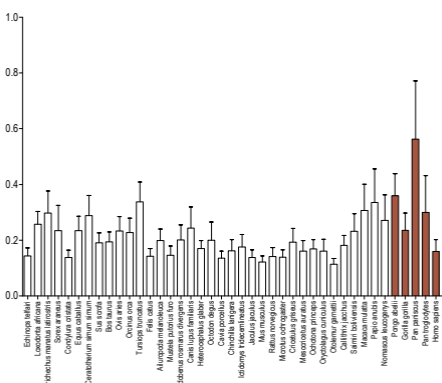

Supplement: Supplementary file 3 [file DataSheet1.PDF]

## SFARI autism genes syndromic

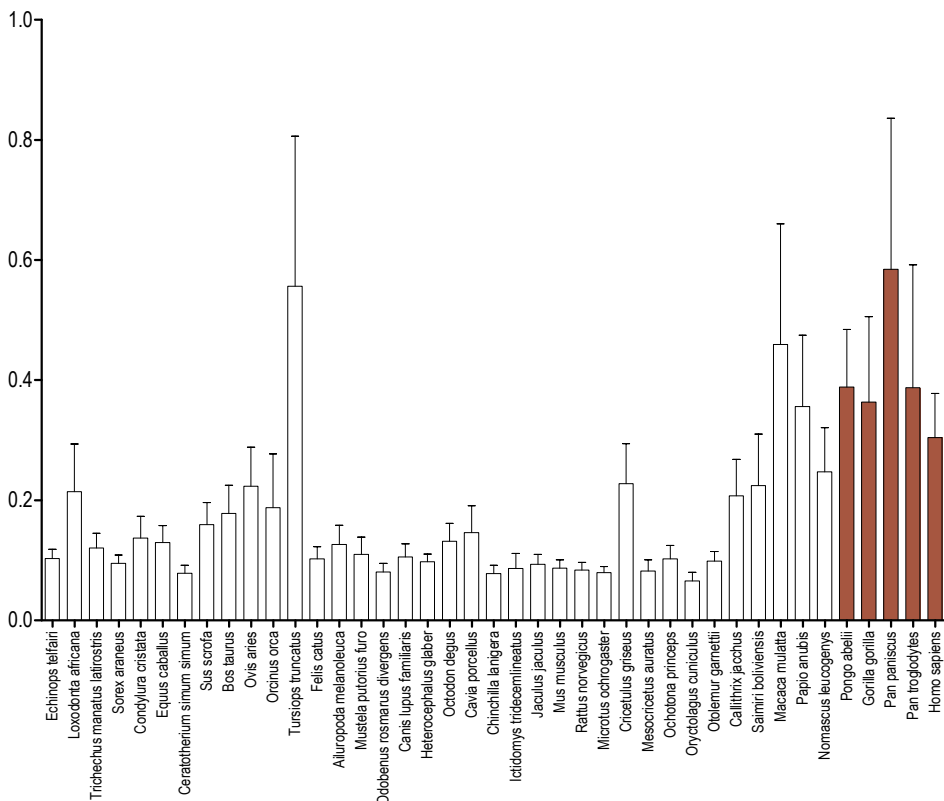

## SFARI autism genes non-syndromic

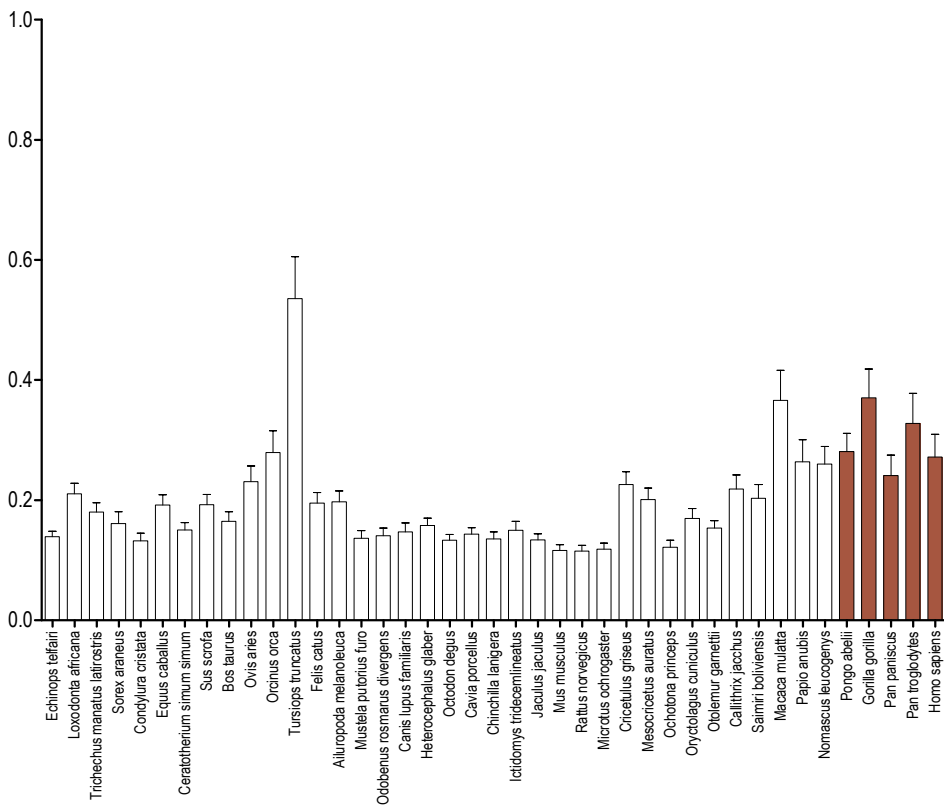

Supplement: Supplementary file 4 [file DataSheet2.PDF]
