# Supplementary material for: Evolutionary conservation in genes underlying human psychiatric disorders
Source: Front Hum Neurosci. 2014 May 6;8:283. doi: 10.3389/fnhum.2014.00283 (PMC4018557; doi:10.3389/fnhum.2014.00283)
Supplement: Supplementary file 6 [file DataSheet4.PDF]

Figure 2A. Genetic Association Database - Schizophrenia

	Loxodonta_africana	Trichechus_manatus_latirostris	Sorex_araneus	Condylura_cristata	Equus_caballus	Ceratotherium_simum_simum	Sus_scrofa	Bos_taurus	Ovis_aries	Orcinus_orca	Tursiops_truncatus	Felis_catus	Ailuropoda_melanoleuca	Mustela_putorius_furo	Odobenus_rosmarus_divergens	Canis_lupus_familiaris	Heterocephalus_glaber	Octodon_degus	Cavia_porcellus	Chinchilla_lanigera	Ictidomys_tridecemlineatus	Jaculus_jaculus	Mus_musculus	Rattus_norvegicus	Microtus_ochrogaster	Cricetulus_griseus	Mesocricetus_auratus	Ochotona_princeps	Oryctolagus_cuniculus	Otolemur_garnettii	Callithrix_jacchus	Saimiri_boliviensis	Macaca_mulatta	Papio_anubis	Nomascus_leucogenys	Pongo_abelii	Gorilla_gorilla	Pan_paniscus	Pan_troglodytes	Homo_sapiens
Echinops_telfairi	ns	ns	ns	ns	ns	ns	ns	ns	ns	ns	****	ns	ns	ns	ns	ns	ns	ns	ns	ns	ns	ns	ns	ns	ns	ns	ns	ns	ns	ns	ns	ns	ns	ns	ns	ns	ns	ns	ns	ns
Loxodonta_africana		ns	ns	ns	ns	ns	ns	ns	ns	ns	****	ns	ns	ns	ns	ns	ns	ns	ns	ns	ns	ns	ns	ns	ns	ns	ns	ns	ns	ns	ns	ns	ns	ns	ns	ns	ns	ns	ns	ns
Trichechus_manatus_latirostris			ns	ns	ns	ns	ns	ns	ns	ns	****	ns	ns	ns	ns	ns	ns	ns	ns	ns	ns	ns	ns	ns	ns	ns	ns	ns	ns	ns	ns	ns	ns	ns	ns	ns	ns	ns	ns	ns
Sorex_araneus				ns	ns	ns	ns	ns	ns	**	****	ns	ns	ns	ns	ns	ns	ns	ns	ns	ns	ns	ns	ns	ns	ns	ns	ns	ns	ns	ns	ns	*	*	ns	**	ns	ns	*	ns
Condylura_cristata					ns	ns	ns	ns	ns	ns	****	ns	ns	ns	ns	ns	ns	ns	ns	ns	ns	ns	ns	ns	ns	ns	ns	ns	ns	ns	ns	ns	ns	ns	ns	ns	ns	ns	ns	ns
Equus_caballus						ns	ns	ns	ns	ns	***	ns	ns	ns	ns	ns	ns	ns	ns	ns	ns	ns	ns	ns	ns	ns	ns	ns	ns	ns	ns	ns	ns	ns	ns	ns	ns	ns	ns	ns
Ceratotherium_simum_simum							ns	ns	ns	ns	****	ns	ns	ns	ns	ns	ns	ns	ns	ns	ns	ns	ns	ns	ns	ns	ns	ns	ns	ns	ns	ns	ns	ns	ns	ns	ns	ns	ns	ns
Sus_scrofa								ns	ns	ns	****	ns	ns	ns	ns	ns	ns	ns	ns	ns	ns	ns	ns	ns	ns	ns	ns	ns	ns	ns	ns	ns	ns	ns	ns	ns	ns	ns	ns	ns
Bos_taurus									ns	ns	****	ns	ns	ns	ns	ns	ns	ns	ns	ns	ns	ns	ns	ns	ns	ns	ns	ns	ns	ns	ns	ns	ns	ns	ns	ns	ns	ns	ns	ns
Ovis_aries										ns	***	ns	ns	ns	ns	ns	ns	ns	ns	ns	ns	ns	ns	ns	ns	ns	ns	ns	ns	ns	ns	ns	ns	ns	ns	ns	ns	ns	ns	ns
Orcinus_orca											ns	ns	ns	**	ns	ns	ns	ns	ns	*	ns	**	**	***	***	ns	ns	***	*	ns	ns	ns	ns	ns	ns	ns	ns	ns	ns	ns
Tursiops_truncatus												****	****	****	****	****	****	****	****	****	****	****	****	****	****	****	****	****	****	****	****	****	ns	ns	***	ns	***	****	ns	****
Felis_catus													ns	ns	ns	ns	ns	ns	ns	ns	ns	ns	ns	ns	ns	ns	ns	ns	ns	ns	ns	ns	ns	ns	ns	ns	ns	ns	ns	ns
Ailuropoda_melanoleuca														ns	ns	ns	ns	ns	ns	ns	ns	ns	ns	ns	ns	ns	ns	ns	ns	ns	ns	ns	ns	ns	ns	ns	ns	ns	ns	ns
Mustela_putorius_furo															ns	ns	ns	ns	ns	ns	ns	ns	ns	ns	ns	ns	ns	ns	ns	ns	ns	ns	*	*	ns	**	ns	ns	**	ns
Odobenus_rosmarus_divergens																ns	ns	ns	ns	ns	ns	ns	ns	ns	ns	ns	ns	ns	ns	ns	ns	ns	ns	ns	ns	ns	ns	ns	ns	ns
Canis_lupus_familiaris																	ns	ns	ns	ns	ns	ns	ns	ns	ns	ns	ns	ns	ns	ns	ns	ns	ns	ns	ns	ns	ns	ns	ns	ns
Heterocephalus_glaber																		ns	ns	ns	ns	ns	ns	ns	ns	ns	ns	ns	ns	ns	ns	ns	ns	ns	ns	ns	ns	ns	ns	ns
Octodon_degus																			ns	ns	ns	ns	ns	ns	ns	ns	ns	ns	ns	ns	ns	ns	ns	ns	ns	ns	ns	ns	ns	ns
Cavia_porcellus																				ns	ns	ns	ns	ns	ns	ns	ns	ns	ns	ns	ns	ns	ns	ns	ns	ns	ns	ns	ns	ns
Chinchilla_lanigera																					ns	ns	ns	ns	ns	ns	ns	ns	ns	ns	ns	ns	ns	ns	ns	ns	ns	ns	ns	ns
Ictidomys_tridecemlineatus																						ns	ns	ns	ns	ns	ns	ns	ns	ns	ns	ns	ns	ns	ns	ns	ns	ns	ns	ns
Jaculus_jaculus																							ns	ns	ns	ns	ns	ns	ns	ns	ns	ns	ns	*	ns	**	ns	ns	*	ns
Mus_musculus																								ns	ns	ns	ns	ns	ns	ns	ns	ns	*	**	ns	**	ns	ns	**	ns
Rattus_norvegicus																									ns	ns	ns	ns	ns	ns	ns	ns	**	**	ns	***	ns	ns	***	ns
Microtus_ochrogaster																										ns	ns	ns	ns	ns	ns	ns	**	**	ns	**	ns	ns	**	ns
Cricetulus_griseus																											ns	ns	ns	ns	ns	ns	ns	ns	ns	ns	ns	ns	ns	ns
Mesocricetus_auratus																												ns	ns	ns	ns	ns	ns	ns	ns	ns	ns	ns	ns	ns
Ochotona_princeps																													ns	ns	ns	ns	**	**	ns	***	ns	ns	**	ns
Oryctolagus_cuniculus																														ns	ns	ns	ns	*	ns	*	ns	ns	*	ns
Otolemur_garnettii																															ns	ns	ns	ns	ns	ns	ns	ns	ns	ns
Callithrix_jacchus																																ns	ns	ns	ns	ns	ns	ns	ns	ns
Saimiri_boliviensis																																	ns	ns	ns	ns	ns	ns	ns	ns
Macaca_mulatta																																		ns	ns	ns	ns	ns	ns	ns
Papio_anubis																																			ns	ns	ns	ns	ns	ns
Nomascus_leucogenys																																				ns	ns	ns	ns	ns
Pongo_abelii																																					ns	ns	ns	ns
Gorilla_gorilla																																						ns	ns	ns
Pan_paniscus																																							ns	ns
Pan_troglodytes																																								ns

Figure 2B. Genetic Association Database - Autism

	Loxodonta_africana	Trichechus_manatus_latirostris	Sorex_araneus	Condylura_cristata	Equus_caballus	Ceratotherium_simum_simum	Sus_scrofa	Bos_taurus	Ovis_aries	Orcinus_orca	Tursiops_truncatus	Felis_catus	Ailuropoda_melanoleuca	Mustela_putorius_furo	Odobenus_rosmarus_divergens	Canis_lupus_familiaris	Heterocephalus_glaber	Octodon_degus	Cavia_porcellus	Chinchilla_lanigera	Ictidomys_tridecemlineatus	Jaculus_jaculus	Mus_musculus	Rattus_norvegicus	Microtus_ochrogaster	Cricetulus_griseus	Mesocricetus_auratus	Ochotona_princeps	Oryctolagus_cuniculus	Otolemur_garnettii	Callithrix_jacchus	Saimiri_boliviensis	Macaca_mulatta	Papio_anubis	Nomascus_leucogenys	Pongo_abelii	Gorilla_gorilla	Pan_paniscus	Pan_troglodytes	Homo_sapiens
Echinops_telfairi	ns	ns	ns	ns	ns	ns	ns	ns	ns	ns	****	ns	ns	ns	ns	ns	ns	ns	ns	ns	ns	ns	ns	ns	ns	ns	ns	ns	ns	ns	ns	ns	ns	ns	ns	ns	ns	ns	ns	ns
Loxodonta_africana		ns	ns	ns	ns	ns	ns	ns	ns	ns	**	ns	ns	ns	ns	ns	ns	ns	ns	ns	ns	ns	ns	ns	ns	ns	ns	ns	ns	ns	ns	ns	ns	ns	ns	ns	ns	ns	ns	ns
Trichechus_manatus_latirostris			ns	ns	ns	ns	ns	ns	ns	ns	**	ns	ns	ns	ns	ns	ns	ns	ns	ns	ns	ns	ns	ns	ns	ns	ns	ns	ns	ns	ns	ns	ns	ns	ns	ns	ns	ns	ns	ns
Sorex_araneus				ns	ns	ns	ns	ns	ns	ns	****	ns	ns	ns	ns	ns	ns	ns	ns	ns	ns	ns	ns	ns	ns	ns	ns	ns	ns	ns	ns	ns	ns	ns	ns	ns	ns	ns	ns	ns
Condylura_cristata					ns	ns	ns	ns	ns	ns	***	ns	ns	ns	ns	ns	ns	ns	ns	ns	ns	ns	ns	ns	ns	ns	ns	ns	ns	ns	ns	ns	ns	ns	ns	ns	ns	ns	ns	ns
Equus_caballus						ns	ns	ns	ns	ns	**	ns	ns	ns	ns	ns	ns	ns	ns	ns	ns	ns	ns	ns	ns	ns	ns	ns	ns	ns	ns	ns	ns	ns	ns	ns	ns	ns	ns	ns
Ceratotherium_simum_simum							ns	ns	ns	ns	****	ns	ns	ns	ns	ns	ns	ns	ns	ns	ns	ns	ns	ns	ns	ns	ns	ns	ns	ns	ns	ns	ns	ns	ns	ns	ns	ns	ns	ns
Sus_scrofa								ns	ns	ns	**	ns	ns	ns	ns	ns	ns	ns	ns	ns	ns	ns	ns	ns	ns	ns	ns	ns	ns	ns	ns	ns	ns	ns	ns	ns	ns	ns	ns	ns
Bos_taurus									ns	ns	**	ns	ns	ns	ns	ns	ns	ns	ns	ns	ns	ns	ns	ns	ns	ns	ns	ns	ns	ns	ns	ns	ns	ns	ns	ns	ns	ns	ns	ns
Ovis_aries										ns	***	ns	ns	ns	ns	ns	ns	ns	ns	ns	ns	ns	ns	ns	ns	ns	ns	ns	ns	ns	ns	ns	ns	ns	ns	ns	ns	ns	ns	ns
Orcinus_orca											**	ns	ns	ns	ns	ns	ns	ns	ns	ns	ns	ns	ns	ns	ns	ns	ns	ns	ns	ns	ns	ns	ns	ns	ns	ns	ns	ns	ns	ns
Tursiops_truncatus												***	*	****	***	***	***	****	****	****	***	****	****	****	****	*	***	****	**	***	****	*	ns	ns	***	ns	ns	****	**	***
Felis_catus													ns	ns	ns	ns	ns	ns	ns	ns	ns	ns	ns	ns	ns	ns	ns	ns	ns	ns	ns	ns	ns	ns	ns	ns	ns	ns	ns	ns
Ailuropoda_melanoleuca														ns	ns	ns	ns	ns	ns	ns	ns	ns	ns	ns	ns	ns	ns	ns	ns	ns	ns	ns	ns	ns	ns	ns	ns	ns	ns	ns
Mustela_putorius_furo															ns	ns	ns	ns	ns	ns	ns	ns	ns	ns	ns	ns	ns	ns	ns	ns	ns	ns	ns	ns	ns	ns	ns	ns	ns	ns
Odobenus_rosmarus_divergens																ns	ns	ns	ns	ns	ns	ns	ns	ns	ns	ns	ns	ns	ns	ns	ns	ns	ns	ns	ns	ns	ns	ns	ns	ns
Canis_lupus_familiaris																	ns	ns	ns	ns	ns	ns	ns	ns	ns	ns	ns	ns	ns	ns	ns	ns	ns	ns	ns	ns	ns	ns	ns	ns
Heterocephalus_glaber																		ns	ns	ns	ns	ns	ns	ns	ns	ns	ns	ns	ns	ns	ns	ns	ns	ns	ns	ns	ns	ns	ns	ns
Octodon_degus																			ns	ns	ns	ns	ns	ns	ns	ns	ns	ns	ns	ns	ns	ns	ns	ns	ns	ns	ns	ns	ns	ns
Cavia_porcellus																				ns	ns	ns	ns	ns	ns	ns	ns	ns	ns	ns	ns	ns	ns	ns	ns	ns	ns	ns	ns	ns
Chinchilla_lanigera																					ns	ns	ns	ns	ns	ns	ns	ns	ns	ns	ns	ns	ns	ns	ns	ns	ns	ns	ns	ns
Ictidomys_tridecemlineatus																						ns	ns	ns	ns	ns	ns	ns	ns	ns	ns	ns	ns	ns	ns	ns	ns	ns	ns	ns
Jaculus_jaculus																							ns	ns	ns	ns	ns	ns	ns	ns	ns	ns	ns	ns	ns	ns	ns	ns	ns	ns
Mus_musculus																								ns	ns	ns	ns	ns	ns	ns	ns	ns	ns	ns	ns	ns	ns	ns	ns	ns
Rattus_norvegicus																									ns	ns	ns	ns	ns	ns	ns	ns	ns	ns	ns	ns	ns	ns	ns	ns
Microtus_ochrogaster																										ns	ns	ns	ns	ns	ns	ns	ns	*	ns	ns	ns	ns	ns	ns
Cricetulus_griseus																											ns	ns	ns	ns	ns	ns	ns	ns	ns	ns	ns	ns	ns	ns
Mesocricetus_auratus																												ns	ns	ns	ns	ns	ns	ns	ns	ns	ns	ns	ns	ns
Ochotona_princeps																													ns	ns	ns	ns	ns	ns	ns	ns	ns	ns	ns	ns
Oryctolagus_cuniculus																														ns	ns	ns	ns	ns	ns	ns	ns	ns	ns	ns
Otolemur_garnettii																															ns	ns	ns	ns	ns	ns	ns	ns	ns	ns
Callithrix_jacchus																																ns	ns	ns	ns	ns	ns	ns	ns	ns
Saimiri_boliviensis																																	ns	ns	ns	ns	ns	ns	ns	ns
Macaca_mulatta																																		ns	ns	ns	ns	ns	ns	ns
Papio_anubis																																			ns	ns	ns	ns	ns	ns
Nomascus_leucogenys																																				ns	ns	ns	ns	ns
Pongo_abelii																																					ns	ns	ns	ns
Gorilla_gorilla																																						ns	ns	ns
Pan_paniscus																																							ns	ns
Pan_troglodytes																																								ns

Figure 3A. Genetic Association Database - ADHD

	Loxodonta_africana	Trichechus_manatus_latirostris	Sorex_araneus	Condylura_cristata	Equus_caballus	Ceratotherium_simum_simum	Sus_scrofa	Bos_taurus	Ovis_aries	Orcinus_orca	Tursiops_truncatus	Felis_catus	Ailuropoda_melanoleuca	Mustela_putorius_furo	Odobenus_rosmarus_divergens	Canis_lupus_familiaris	Heterocephalus_glaber	Octodon_degus	Cavia_porcellus	Chinchilla_lanigera	Ictidomys_tridecemlineatus	Jaculus_jaculus	Mus_musculus	Rattus_norvegicus	Microtus_ochrogaster	Cricetulus_griseus	Mesocricetus_auratus	Ochotona_princeps	Oryctolagus_cuniculus	Otolemur_garnettii	Callithrix_jacchus	Saimiri_boliviensis	Macaca_mulatta	Papio_anubis	Nomascus_leucogenys	Pongo_abelii	Gorilla_gorilla	Pan_paniscus	Pan_troglodytes	Homo_sapiens
Echinops_telfairi	ns	ns	ns	ns	ns	ns	ns	ns	ns	ns	****	ns	ns	ns	ns	ns	ns	ns	ns	ns	ns	ns	ns	ns	ns	ns	ns	ns	ns	ns	ns	ns	ns	*	ns	ns	ns	ns	*	ns
Loxodonta_africana		ns	ns	ns	ns	ns	ns	ns	ns	ns	****	ns	ns	ns	ns	ns	ns	ns	ns	ns	ns	ns	ns	ns	ns	ns	ns	ns	ns	ns	ns	ns	ns	ns	ns	ns	ns	ns	ns	ns
Trichechus_manatus_latirostris			ns	ns	ns	ns	ns	ns	ns	ns	****	ns	ns	ns	ns	ns	ns	ns	ns	ns	ns	ns	ns	ns	ns	ns	ns	ns	ns	ns	ns	ns	ns	ns	ns	ns	ns	ns	ns	ns
Sorex_araneus				ns	ns	ns	ns	ns	ns	ns	****	ns	ns	ns	ns	ns	ns	ns	ns	ns	ns	ns	ns	ns	ns	ns	ns	ns	ns	ns	ns	ns	**	***	ns	ns	ns	ns	**	ns
Condylura_cristata					ns	ns	ns	ns	ns	ns	****	ns	ns	ns	ns	ns	ns	ns	ns	ns	ns	ns	ns	ns	ns	ns	ns	ns	ns	ns	ns	ns	*	**	ns	ns	ns	ns	**	ns
Equus_caballus						ns	ns	ns	ns	ns	****	ns	ns	ns	ns	ns	ns	ns	ns	ns	ns	ns	ns	ns	ns	ns	ns	ns	ns	ns	ns	ns	ns	ns	ns	ns	ns	ns	ns	ns
Ceratotherium_simum_simum							ns	ns	ns	ns	****	ns	ns	ns	ns	ns	ns	ns	ns	ns	ns	ns	ns	ns	ns	ns	ns	ns	ns	ns	ns	ns	ns	*	ns	ns	ns	ns	ns	ns
Sus_scrofa								ns	ns	ns	****	ns	ns	ns	ns	ns	ns	ns	ns	ns	ns	ns	ns	ns	ns	ns	ns	ns	ns	ns	ns	ns	ns	ns	ns	ns	ns	ns	ns	ns
Bos_taurus									ns	ns	****	ns	ns	ns	ns	ns	ns	ns	ns	ns	ns	ns	ns	ns	ns	ns	ns	ns	ns	ns	ns	ns	ns	ns	ns	ns	ns	ns	ns	ns
Ovis_aries										ns	****	ns	ns	ns	ns	ns	ns	ns	ns	ns	ns	ns	ns	ns	ns	ns	ns	ns	ns	ns	ns	ns	ns	ns	ns	ns	ns	ns	ns	ns
Orcinus_orca											***	ns	ns	ns	ns	ns	ns	ns	ns	ns	ns	ns	*	*	*	ns	ns	ns	ns	ns	ns	ns	ns	ns	ns	ns	ns	ns	ns	ns
Tursiops_truncatus												****	****	****	****	****	****	****	****	****	****	****	****	****	****	****	****	****	****	****	**	****	*	ns	****	****	***	***	ns	****
Felis_catus													ns	ns	ns	ns	ns	ns	ns	ns	ns	ns	ns	ns	ns	ns	ns	ns	ns	ns	ns	ns	ns	ns	ns	ns	ns	ns	ns	ns
Ailuropoda_melanoleuca														ns	ns	ns	ns	ns	ns	ns	ns	ns	ns	ns	ns	ns	ns	ns	ns	ns	ns	ns	ns	ns	ns	ns	ns	ns	ns	ns
Mustela_putorius_furo															ns	ns	ns	ns	ns	ns	ns	ns	ns	ns	ns	ns	ns	ns	ns	ns	ns	ns	**	***	ns	ns	ns	ns	***	ns
Odobenus_rosmarus_divergens																ns	ns	ns	ns	ns	ns	ns	ns	ns	ns	ns	ns	ns	ns	ns	ns	ns	ns	**	ns	ns	ns	ns	*	ns
Canis_lupus_familiaris																	ns	ns	ns	ns	ns	ns	ns	ns	ns	ns	ns	ns	ns	ns	ns	ns	*	**	ns	ns	ns	ns	*	ns
Heterocephalus_glaber																		ns	ns	ns	ns	ns	ns	ns	ns	ns	ns	ns	ns	ns	ns	ns	ns	*	ns	ns	ns	ns	ns	ns
Octodon_degus																			ns	ns	ns	ns	ns	ns	ns	ns	ns	ns	ns	ns	ns	ns	*	**	ns	ns	ns	ns	**	ns
Cavia_porcellus																				ns	ns	ns	ns	ns	ns	ns	ns	ns	ns	ns	ns	ns	ns	ns	ns	ns	ns	ns	ns	ns
Chinchilla_lanigera																					ns	ns	ns	ns	ns	ns	ns	ns	ns	ns	ns	ns	*	**	ns	ns	ns	ns	*	ns
Ictidomys_tridecemlineatus																						ns	ns	ns	ns	ns	ns	ns	ns	ns	ns	ns	**	****	ns	ns	ns	ns	***	ns
Jaculus_jaculus																							ns	ns	ns	ns	ns	ns	ns	ns	ns	ns	*	**	ns	ns	ns	ns	*	ns
Mus_musculus																								ns	ns	ns	ns	ns	ns	ns	**	ns	***	****	ns	ns	**	*	****	ns
Rattus_norvegicus																									ns	ns	ns	ns	ns	ns	*	ns	***	****	ns	ns	*	ns	***	ns
Microtus_ochrogaster																										ns	ns	ns	ns	ns	*	ns	***	****	ns	ns	*	ns	***	ns
Cricetulus_griseus																											ns	ns	ns	ns	ns	ns	ns	ns	ns	ns	ns	ns	ns	ns
Mesocricetus_auratus																												ns	ns	ns	ns	ns	ns	ns	ns	ns	ns	ns	ns	ns
Ochotona_princeps																													ns	ns	ns	ns	**	***	ns	ns	ns	ns	**	ns
Oryctolagus_cuniculus																														ns	ns	ns	ns	**	ns	ns	ns	ns	*	ns
Otolemur_garnettii																															ns	ns	ns	*	ns	ns	ns	ns	ns	ns
Callithrix_jacchus																																ns	ns	ns	ns	ns	ns	ns	ns	ns
Saimiri_boliviensis																																	ns	ns	ns	ns	ns	ns	ns	ns
Macaca_mulatta																																		ns	ns	ns	ns	ns	ns	ns
Papio_anubis																																			ns	ns	ns	ns	ns	*
Nomascus_leucogenys																																				ns	ns	ns	ns	ns
Pongo_abelii																																					ns	ns	ns	ns
Gorilla_gorilla																																						ns	ns	ns
Pan_paniscus																																							ns	ns
Pan_troglodytes																																								ns

Figure 3B. Genetic Association Database - Bipolar

	Loxodonta_africana	Trichechus_manatus_latirostris	Sorex_araneus	Condylura_cristata	Equus_caballus	Ceratotherium_simum_simum	Sus_scrofa	Bos_taurus	Ovis_aries	Orcinus_orca	Tursiops_truncatus	Felis_catus	Ailuropoda_melanoleuca	Mustela_putorius_furo	Odobenus_rosmarus_divergens	Canis_lupus_familiaris	Heterocephalus_glaber	Octodon_degus	Cavia_porcellus	Chinchilla_lanigera	Ictidomys_tridecemlineatus	Jaculus_jaculus	Mus_musculus	Rattus_norvegicus	Microtus_ochrogaster	Cricetulus_griseus	Mesocricetus_auratus	Ochotona_princeps	Oryctolagus_cuniculus	Otolemur_garnettii	Callithrix_jacchus	Saimiri_boliviensis	Macaca_mulatta	Papio_anubis	Nomascus_leucogenys	Pongo_abelii	Gorilla_gorilla	Pan_paniscus	Pan_troglodytes	Homo_sapiens
Echinops_telfairi	ns	ns	ns	ns	ns	ns	ns	ns	ns	ns	****	ns	ns	ns	ns	ns	ns	ns	ns	ns	ns	ns	ns	ns	ns	ns	ns	ns	ns	ns	ns	ns	*	ns	ns	ns	ns	ns	****	ns
Loxodonta_africana		ns	ns	ns	ns	ns	ns	ns	ns	ns	***	ns	ns	ns	ns	ns	ns	ns	ns	ns	ns	ns	ns	ns	ns	ns	ns	ns	ns	ns	ns	ns	ns	ns	ns	ns	ns	ns	ns	ns
Trichechus_manatus_latirostris			ns	ns	ns	ns	ns	ns	ns	ns	****	ns	ns	ns	ns	ns	ns	ns	ns	ns	ns	ns	ns	ns	ns	ns	ns	ns	ns	ns	ns	ns	ns	ns	ns	ns	ns	ns	**	ns
Sorex_araneus				ns	ns	ns	ns	ns	ns	ns	****	ns	ns	ns	ns	ns	ns	ns	ns	ns	ns	ns	ns	ns	ns	ns	ns	ns	ns	ns	ns	ns	***	**	ns	ns	ns	ns	****	ns
Condylura_cristata					ns	ns	ns	ns	ns	ns	****	ns	ns	ns	ns	ns	ns	ns	ns	ns	ns	ns	ns	ns	ns	ns	ns	ns	ns	ns	ns	ns	**	*	ns	ns	ns	ns	****	ns
Equus_caballus						ns	ns	ns	ns	ns	****	ns	ns	ns	ns	ns	ns	ns	ns	ns	ns	ns	ns	ns	ns	ns	ns	ns	ns	ns	ns	ns	ns	ns	ns	ns	ns	ns	ns	ns
Ceratotherium_simum_simum							ns	ns	ns	ns	****	ns	ns	ns	ns	ns	ns	ns	ns	ns	ns	ns	ns	ns	ns	ns	ns	ns	ns	ns	ns	ns	ns	ns	ns	ns	ns	ns	***	ns
Sus_scrofa								ns	ns	ns	****	ns	ns	ns	ns	ns	ns	ns	ns	ns	ns	ns	ns	ns	ns	ns	ns	ns	ns	ns	ns	ns	ns	ns	ns	ns	ns	ns	*	ns
Bos_taurus									ns	ns	****	ns	ns	ns	ns	ns	ns	ns	ns	ns	ns	ns	ns	ns	ns	ns	ns	ns	ns	ns	ns	ns	**	*	ns	ns	ns	ns	****	ns
Ovis_aries										ns	****	ns	ns	ns	ns	ns	ns	ns	ns	ns	ns	ns	ns	ns	ns	ns	ns	ns	ns	ns	ns	ns	ns	ns	ns	ns	ns	ns	*	ns
Orcinus_orca											**	ns	ns	ns	ns	ns	ns	ns	ns	ns	ns	ns	*	*	**	ns	ns	ns	ns	ns	ns	ns	ns	ns	ns	ns	ns	ns	ns	ns
Tursiops_truncatus												****	***	****	****	****	****	****	****	****	****	****	****	****	****	**	****	****	****	****	****	***	ns	ns	****	ns	**	****	ns	****
Felis_catus													ns	ns	ns	ns	ns	ns	ns	ns	ns	ns	ns	ns	ns	ns	ns	ns	ns	ns	ns	ns	ns	ns	ns	ns	ns	ns	***	ns
Ailuropoda_melanoleuca														ns	ns	ns	ns	ns	ns	ns	ns	ns	ns	ns	ns	ns	ns	ns	ns	ns	ns	ns	ns	ns	ns	ns	ns	ns	ns	ns
Mustela_putorius_furo															ns	ns	ns	ns	ns	ns	ns	ns	ns	ns	ns	ns	ns	ns	ns	ns	ns	ns	*	ns	ns	ns	ns	ns	****	ns
Odobenus_rosmarus_divergens																ns	ns	ns	ns	ns	ns	ns	ns	ns	ns	ns	ns	ns	ns	ns	ns	ns	**	*	ns	ns	ns	ns	****	ns
Canis_lupus_familiaris																	ns	ns	ns	ns	ns	ns	ns	ns	ns	ns	ns	ns	ns	ns	ns	ns	*	ns	ns	ns	ns	ns	****	ns
Heterocephalus_glaber																		ns	ns	ns	ns	ns	ns	ns	ns	ns	ns	ns	ns	ns	ns	ns	**	*	ns	ns	ns	ns	****	ns
Octodon_degus																			ns	ns	ns	ns	ns	ns	ns	ns	ns	ns	ns	ns	ns	ns	****	***	ns	*	ns	ns	****	ns
Cavia_porcellus																				ns	ns	ns	ns	ns	ns	ns	ns	ns	ns	ns	ns	ns	**	*	ns	ns	ns	ns	****	ns
Chinchilla_lanigera																					ns	ns	ns	ns	ns	ns	ns	ns	ns	ns	ns	ns	*	ns	ns	ns	ns	ns	****	ns
Ictidomys_tridecemlineatus																						ns	ns	ns	ns	ns	ns	ns	ns	ns	ns	ns	**	*	ns	ns	ns	ns	****	ns
Jaculus_jaculus																							ns	ns	ns	ns	ns	ns	ns	ns	ns	ns	**	*	ns	ns	ns	ns	****	ns
Mus_musculus																								ns	ns	*	ns	ns	ns	ns	ns	ns	****	***	ns	**	*	ns	****	ns
Rattus_norvegicus																									ns	*	ns	ns	ns	ns	ns	ns	****	****	ns	**	*	ns	****	ns
Microtus_ochrogaster																										*	ns	ns	ns	ns	ns	ns	****	****	ns	**	*	ns	****	ns
Cricetulus_griseus																											ns	ns	ns	ns	ns	ns	ns	ns	ns	ns	ns	ns	ns	ns
Mesocricetus_auratus																												ns	ns	ns	ns	ns	ns	ns	ns	ns	ns	ns	****	ns
Ochotona_princeps																													ns	ns	ns	ns	**	*	ns	ns	ns	ns	****	ns
Oryctolagus_cuniculus																														ns	ns	ns	***	**	ns	ns	ns	ns	****	ns
Otolemur_garnettii																															ns	ns	*	ns	ns	ns	ns	ns	****	ns
Callithrix_jacchus																																ns	ns	ns	ns	ns	ns	ns	*	ns
Saimiri_boliviensis																																	ns	ns	ns	ns	ns	ns	ns	ns
Macaca_mulatta																																		ns	ns	ns	ns	ns	ns	ns
Papio_anubis																																			ns	ns	ns	ns	ns	ns
Nomascus_leucogenys																																				ns	ns	ns	*	ns
Pongo_abelii																																					ns	ns	ns	ns
Gorilla_gorilla																																						ns	ns	ns
Pan_paniscus																																							*	ns
Pan_troglodytes																																								***

Figure 3C. Genetic Association Database - Depression

	Loxodonta_africana	Trichechus_manatus_latirostris	Sorex_araneus	Condylura_cristata	Equus_caballus	Ceratotherium_simum_simum	Sus_scrofa	Bos_taurus	Ovis_aries	Orcinus_orca	Tursiops_truncatus	Felis_catus	Ailuropoda_melanoleuca	Mustela_putorius_furo	Odobenus_rosmarus_divergens	Canis_lupus_familiaris	Heterocephalus_glaber	Octodon_degus	Cavia_porcellus	Chinchilla_lanigera	Ictidomys_tridecemlineatus	Jaculus_jaculus	Mus_musculus	Rattus_norvegicus	Microtus_ochrogaster	Cricetulus_griseus	Mesocricetus_auratus	Ochotona_princeps	Oryctolagus_cuniculus	Otolemur_garnettii	Callithrix_jacchus	Saimiri_boliviensis	Macaca_mulatta	Papio_anubis	Nomascus_leucogenys	Pongo_abelii	Gorilla_gorilla	Pan_paniscus	Pan_troglodytes	Homo_sapiens
Echinops_telfairi	ns	ns	ns	ns	ns	ns	ns	ns	ns	ns	***	ns	ns	ns	ns	ns	ns	ns	ns	ns	ns	ns	ns	ns	ns	ns	ns	ns	ns	ns	ns	ns	ns	ns	ns	ns	ns	ns	ns	ns
Loxodonta_africana		ns	ns	ns	ns	ns	ns	ns	ns	ns	**	ns	ns	ns	ns	ns	ns	ns	ns	ns	ns	ns	ns	ns	ns	ns	ns	ns	ns	ns	ns	ns	ns	ns	ns	ns	ns	ns	ns	ns
Trichechus_manatus_latirostris			ns	ns	ns	ns	ns	ns	ns	ns	ns	ns	ns	ns	ns	ns	ns	ns	ns	ns	ns	ns	ns	ns	ns	ns	ns	ns	ns	ns	ns	ns	ns	ns	ns	ns	ns	ns	ns	ns
Sorex_araneus				ns	ns	ns	ns	ns	ns	ns	****	ns	ns	ns	ns	ns	ns	ns	ns	ns	ns	ns	ns	ns	ns	ns	ns	ns	ns	ns	ns	ns	ns	ns	ns	*	ns	ns	ns	ns
Condylura_cristata					ns	ns	ns	ns	ns	ns	****	ns	ns	ns	ns	ns	ns	ns	ns	ns	ns	ns	ns	ns	ns	ns	ns	ns	ns	ns	ns	ns	ns	ns	ns	ns	ns	ns	ns	ns
Equus_caballus						ns	ns	ns	ns	ns	*	ns	ns	ns	ns	ns	ns	ns	ns	ns	ns	ns	ns	ns	ns	ns	ns	ns	ns	ns	ns	ns	ns	ns	ns	ns	ns	ns	ns	ns
Ceratotherium_simum_simum							ns	ns	ns	ns	**	ns	ns	ns	ns	ns	ns	ns	ns	ns	ns	ns	ns	ns	ns	ns	ns	ns	ns	ns	ns	ns	ns	ns	ns	ns	ns	ns	ns	ns
Sus_scrofa								ns	ns	ns	*	ns	ns	ns	ns	ns	ns	ns	ns	ns	ns	ns	ns	ns	ns	ns	ns	ns	ns	ns	ns	ns	ns	ns	ns	ns	ns	ns	ns	ns
Bos_taurus									ns	ns	**	ns	ns	ns	ns	ns	ns	ns	ns	ns	ns	ns	ns	ns	ns	ns	ns	ns	ns	ns	ns	ns	ns	ns	ns	ns	ns	ns	ns	ns
Ovis_aries										ns	*	ns	ns	ns	ns	ns	ns	ns	ns	ns	ns	ns	ns	ns	ns	ns	ns	ns	ns	ns	ns	ns	ns	ns	ns	ns	ns	ns	ns	ns
Orcinus_orca											ns	ns	ns	ns	ns	ns	ns	ns	ns	ns	ns	ns	ns	ns	ns	ns	ns	ns	ns	ns	ns	ns	ns	ns	ns	ns	ns	ns	ns	ns
Tursiops_truncatus												**	ns	****	*	***	***	***	**	***	****	****	****	****	****	ns	***	***	**	****	ns	**	ns	ns	ns	ns	ns	*	ns	***
Felis_catus													ns	ns	ns	ns	ns	ns	ns	ns	ns	ns	ns	ns	ns	ns	ns	ns	ns	ns	ns	ns	ns	ns	ns	ns	ns	ns	ns	ns
Ailuropoda_melanoleuca														ns	ns	ns	ns	ns	ns	ns	ns	ns	ns	ns	ns	ns	ns	ns	ns	ns	ns	ns	ns	ns	ns	ns	ns	ns	ns	ns
Mustela_putorius_furo															ns	ns	ns	ns	ns	ns	ns	ns	ns	ns	ns	ns	ns	ns	ns	ns	ns	ns	ns	ns	ns	*	ns	ns	ns	ns
Odobenus_rosmarus_divergens																ns	ns	ns	ns	ns	ns	ns	ns	ns	ns	ns	ns	ns	ns	ns	ns	ns	ns	ns	ns	ns	ns	ns	ns	ns
Canis_lupus_familiaris																	ns	ns	ns	ns	ns	ns	ns	ns	ns	ns	ns	ns	ns	ns	ns	ns	ns	ns	ns	ns	ns	ns	ns	ns
Heterocephalus_glaber																		ns	ns	ns	ns	ns	ns	ns	ns	ns	ns	ns	ns	ns	ns	ns	ns	ns	ns	ns	ns	ns	ns	ns
Octodon_degus																			ns	ns	ns	ns	ns	ns	ns	ns	ns	ns	ns	ns	ns	ns	ns	ns	ns	ns	ns	ns	ns	ns
Cavia_porcellus																				ns	ns	ns	ns	ns	ns	ns	ns	ns	ns	ns	ns	ns	ns	ns	ns	ns	ns	ns	ns	ns
Chinchilla_lanigera																					ns	ns	ns	ns	ns	ns	ns	ns	ns	ns	ns	ns	ns	ns	ns	ns	ns	ns	ns	ns
Ictidomys_tridecemlineatus																						ns	ns	ns	ns	ns	ns	ns	ns	ns	ns	ns	ns	ns	ns	ns	ns	ns	ns	ns
Jaculus_jaculus																							ns	ns	ns	ns	ns	ns	ns	ns	ns	ns	ns	ns	ns	ns	ns	ns	ns	ns
Mus_musculus																								ns	ns	ns	ns	ns	ns	ns	ns	ns	ns	ns	ns	**	ns	ns	ns	ns
Rattus_norvegicus																									ns	ns	ns	ns	ns	ns	ns	ns	ns	ns	ns	*	ns	ns	ns	ns
Microtus_ochrogaster																										ns	ns	ns	ns	ns	ns	ns	ns	ns	ns	**	ns	ns	ns	ns
Cricetulus_griseus																											ns	ns	ns	ns	ns	ns	ns	ns	ns	ns	ns	ns	ns	ns
Mesocricetus_auratus																												ns	ns	ns	ns	ns	ns	ns	ns	ns	ns	ns	ns	ns
Ochotona_princeps																													ns	ns	ns	ns	ns	ns	ns	ns	ns	ns	ns	ns
Oryctolagus_cuniculus																														ns	ns	ns	ns	ns	ns	ns	ns	ns	ns	ns
Otolemur_garnettii																															ns	ns	ns	ns	ns	*	ns	ns	ns	ns
Callithrix_jacchus																																ns	ns	ns	ns	ns	ns	ns	ns	ns
Saimiri_boliviensis																																	ns	ns	ns	ns	ns	ns	ns	ns
Macaca_mulatta																																		ns	ns	ns	ns	ns	ns	ns
Papio_anubis																																			ns	ns	ns	ns	ns	ns
Nomascus_leucogenys																																				ns	ns	ns	ns	ns
Pongo_abelii																																					ns	ns	ns	ns
Gorilla_gorilla																																						ns	ns	ns
Pan_paniscus																																							ns	ns
Pan_troglodytes																																								ns

Figure 3D. Genetic Association Database - Anxiety

	Loxodonta_africana	Trichechus_manatus_latirostris	Sorex_araneus	Condylura_cristata	Equus_caballus	Ceratotherium_simum_simum	Sus_scrofa	Bos_taurus	Ovis_aries	Orcinus_orca	Tursiops_truncatus	Felis_catus	Ailuropoda_melanoleuca	Mustela_putorius_furo	Odobenus_rosmarus_divergens	Canis_lupus_familiaris	Heterocephalus_glaber	Octodon_degus	Cavia_porcellus	Chinchilla_lanigera	Ictidomys_tridecemlineatus	Jaculus_jaculus	Mus_musculus	Rattus_norvegicus	Microtus_ochrogaster	Cricetulus_griseus	Mesocricetus_auratus	Ochotona_princeps	Oryctolagus_cuniculus	Otolemur_garnettii	Callithrix_jacchus	Saimiri_boliviensis	Macaca_mulatta	Papio_anubis	Nomascus_leucogenys	Pongo_abelii	Gorilla_gorilla	Pan_paniscus	Pan_troglodytes	Homo_sapiens
Echinops_telfairi	ns	ns	ns	ns	ns	ns	ns	ns	ns	ns	ns	ns	ns	ns	ns	ns	ns	ns	ns	ns	ns	ns	ns	ns	ns	ns	ns	ns	ns	ns	ns	ns	ns	ns	ns	ns	ns	ns	ns	ns
Loxodonta_africana		ns	ns	ns	ns	ns	ns	ns	ns	ns	ns	ns	ns	ns	ns	ns	ns	ns	ns	ns	ns	ns	ns	ns	ns	ns	ns	ns	ns	ns	ns	ns	ns	ns	ns	ns	ns	ns	ns	ns
Trichechus_manatus_latirostris			ns	ns	ns	ns	ns	ns	ns	ns	ns	ns	ns	ns	ns	ns	ns	ns	ns	ns	ns	ns	ns	ns	ns	ns	ns	ns	ns	ns	ns	ns	ns	ns	ns	ns	ns	ns	ns	ns
Sorex_araneus				ns	ns	ns	ns	ns	ns	ns	ns	ns	ns	ns	ns	ns	ns	ns	ns	ns	ns	ns	ns	ns	ns	ns	ns	ns	ns	ns	ns	ns	ns	ns	ns	ns	ns	ns	ns	ns
Condylura_cristata					ns	ns	ns	ns	ns	ns	ns	ns	ns	ns	ns	ns	ns	ns	ns	ns	ns	ns	ns	ns	ns	ns	ns	ns	ns	ns	ns	ns	ns	ns	ns	ns	ns	ns	ns	ns
Equus_caballus						ns	ns	ns	ns	ns	ns	ns	ns	ns	ns	ns	ns	ns	ns	ns	ns	ns	ns	ns	ns	ns	ns	ns	ns	ns	ns	ns	ns	ns	ns	ns	ns	ns	ns	ns
Ceratotherium_simum_simum							ns	ns	ns	ns	ns	ns	ns	ns	ns	ns	ns	ns	ns	ns	ns	ns	ns	ns	ns	ns	ns	ns	ns	ns	ns	ns	ns	ns	ns	ns	ns	ns	ns	ns
Sus_scrofa								ns	ns	ns	ns	ns	ns	ns	ns	ns	ns	ns	ns	ns	ns	ns	ns	ns	ns	ns	ns	ns	ns	ns	ns	ns	ns	ns	ns	ns	ns	ns	ns	ns
Bos_taurus									ns	ns	ns	ns	ns	ns	ns	ns	ns	ns	ns	ns	ns	ns	ns	ns	ns	ns	ns	ns	ns	ns	ns	ns	ns	ns	ns	ns	ns	ns	ns	ns
Ovis_aries										ns	ns	ns	ns	ns	ns	ns	ns	ns	ns	ns	ns	ns	ns	ns	ns	ns	ns	ns	ns	ns	ns	ns	ns	ns	ns	ns	ns	ns	ns	ns
Orcinus_orca											ns	ns	ns	ns	ns	ns	ns	ns	ns	ns	ns	ns	ns	ns	ns	ns	ns	ns	ns	ns	ns	ns	ns	ns	ns	ns	ns	ns	ns	ns
Tursiops_truncatus												ns	ns	ns	ns	ns	ns	ns	ns	ns	ns	ns	ns	ns	ns	ns	ns	ns	ns	ns	ns	ns	ns	ns	ns	ns	ns	ns	ns	ns
Felis_catus													ns	ns	ns	ns	ns	ns	ns	ns	ns	ns	ns	ns	ns	ns	ns	ns	ns	ns	ns	ns	ns	ns	ns	ns	ns	ns	ns	ns
Ailuropoda_melanoleuca														ns	ns	ns	ns	ns	ns	ns	ns	ns	ns	ns	ns	ns	ns	ns	ns	ns	ns	ns	ns	ns	ns	ns	ns	ns	ns	ns
Mustela_putorius_furo															ns	ns	ns	ns	ns	ns	ns	ns	ns	ns	ns	ns	ns	ns	ns	ns	ns	ns	ns	ns	ns	ns	ns	ns	ns	ns
Odobenus_rosmarus_divergens																ns	ns	ns	ns	ns	ns	ns	ns	ns	ns	ns	ns	ns	ns	ns	ns	ns	ns	ns	ns	ns	ns	ns	ns	ns
Canis_lupus_familiaris																	ns	ns	ns	ns	ns	ns	ns	ns	ns	ns	ns	ns	ns	ns	ns	ns	ns	ns	ns	ns	ns	ns	ns	ns
Heterocephalus_glaber																		ns	ns	ns	ns	ns	ns	ns	ns	ns	ns	ns	ns	ns	ns	ns	ns	ns	ns	ns	ns	ns	ns	ns
Octodon_degus																			ns	ns	ns	ns	ns	ns	ns	ns	ns	ns	ns	ns	ns	ns	ns	ns	ns	ns	ns	ns	ns	ns
Cavia_porcellus																				ns	ns	ns	ns	ns	ns	ns	ns	ns	ns	ns	ns	ns	ns	ns	ns	ns	ns	ns	ns	ns
Chinchilla_lanigera																					ns	ns	ns	ns	ns	ns	ns	ns	ns	ns	ns	ns	ns	ns	ns	ns	ns	ns	ns	ns
Ictidomys_tridecemlineatus																						ns	ns	ns	ns	ns	ns	ns	ns	ns	ns	ns	ns	ns	ns	ns	ns	ns	ns	ns
Jaculus_jaculus																							ns	ns	ns	ns	ns	ns	ns	ns	ns	ns	ns	ns	ns	ns	ns	ns	ns	ns
Mus_musculus																								ns	ns	ns	ns	ns	ns	ns	ns	ns	ns	ns	ns	ns	ns	ns	ns	ns
Rattus_norvegicus																									ns	ns	ns	ns	ns	ns	ns	ns	ns	ns	ns	ns	ns	ns	ns	ns
Microtus_ochrogaster																										ns	ns	ns	ns	ns	ns	ns	ns	ns	ns	ns	ns	ns	ns	ns
Cricetulus_griseus																											ns	ns	ns	ns	ns	ns	ns	ns	ns	ns	ns	ns	ns	ns
Mesocricetus_auratus																												ns	ns	ns	ns	ns	ns	ns	ns	ns	ns	ns	ns	ns
Ochotona_princeps																													ns	ns	ns	ns	ns	ns	ns	ns	ns	ns	ns	ns
Oryctolagus_cuniculus																														ns	ns	ns	ns	ns	ns	ns	ns	ns	ns	ns
Otolemur_garnettii																															ns	ns	ns	ns	ns	ns	ns	ns	ns	ns
Callithrix_jacchus																																ns	ns	ns	ns	ns	ns	ns	ns	ns
Saimiri_boliviensis																																	ns	ns	ns	ns	ns	ns	ns	ns
Macaca_mulatta																																		ns	ns	ns	ns	ns	ns	ns
Papio_anubis																																			ns	ns	ns	ns	ns	ns
Nomascus_leucogenys																																				ns	ns	ns	ns	ns
Pongo_abelii																																					ns	ns	ns	ns
Gorilla_gorilla																																						ns	ns	ns
Pan_paniscus																																							ns	ns
Pan_troglodytes																																								ns

Figure 3E. Genetic Association Database - Addiction

	Loxodonta_africana	Trichechus_manatus_latirostris	Sorex_araneus	Condylura_cristata	Equus_caballus	Ceratotherium_simum_simum	Sus_scrofa	Bos_taurus	Ovis_aries	Orcinus_orca	Tursiops_truncatus	Felis_catus	Ailuropoda_melanoleuca	Mustela_putorius_furo	Odobenus_rosmarus_divergens	Canis_lupus_familiaris	Heterocephalus_glaber	Octodon_degus	Cavia_porcellus	Chinchilla_lanigera	Ictidomys_tridecemlineatus	Jaculus_jaculus	Mus_musculus	Rattus_norvegicus	Microtus_ochrogaster	Cricetulus_griseus	Mesocricetus_auratus	Ochotona_princeps	Oryctolagus_cuniculus	Otolemur_garnettii	Callithrix_jacchus	Saimiri_boliviensis	Macaca_mulatta	Papio_anubis	Nomascus_leucogenys	Pongo_abelii	Gorilla_gorilla	Pan_paniscus	Pan_troglodytes	Homo_sapiens
Echinops_telfairi	ns	ns	ns	ns	ns	ns	ns	ns	ns	****	****	ns	ns	ns	ns	ns	ns	ns	ns	ns	ns	ns	ns	ns	ns	ns	ns	ns	ns	ns	ns	ns	**	ns	ns	*	ns	****	ns	ns
Loxodonta_africana		ns	ns	ns	ns	ns	ns	ns	ns	ns	****	ns	ns	ns	ns	ns	ns	ns	ns	ns	ns	ns	**	*	*	ns	ns	ns	ns	ns	ns	ns	ns	ns	ns	ns	ns	ns	ns	ns
Trichechus_manatus_latirostris			ns	ns	ns	ns	ns	ns	ns	ns	****	ns	ns	ns	ns	ns	ns	ns	ns	ns	ns	ns	ns	ns	ns	ns	ns	ns	ns	ns	ns	ns	ns	ns	ns	ns	ns	ns	ns	ns
Sorex_araneus				ns	ns	ns	ns	ns	*	****	****	ns	ns	ns	ns	ns	ns	ns	ns	ns	ns	ns	ns	ns	ns	ns	ns	ns	ns	ns	**	ns	****	*	ns	****	**	****	***	ns
Condylura_cristata					ns	ns	ns	ns	ns	****	****	ns	ns	ns	ns	ns	ns	ns	ns	ns	ns	ns	ns	ns	ns	ns	ns	ns	ns	ns	*	ns	****	*	ns	***	**	****	**	ns
Equus_caballus						ns	ns	ns	ns	*	****	ns	ns	ns	ns	ns	ns	ns	ns	ns	ns	ns	ns	ns	ns	ns	ns	ns	ns	ns	ns	ns	ns	ns	ns	ns	ns	*	ns	ns
Ceratotherium_simum_simum							ns	ns	ns	***	****	ns	ns	ns	ns	ns	ns	ns	ns	ns	ns	ns	ns	ns	ns	ns	ns	ns	ns	ns	ns	ns	ns	ns	ns	ns	ns	**	ns	ns
Sus_scrofa								ns	ns	*	****	ns	ns	ns	ns	ns	ns	ns	ns	ns	ns	ns	ns	ns	ns	ns	ns	ns	ns	ns	ns	ns	ns	ns	ns	ns	ns	*	ns	ns
Bos_taurus									ns	****	****	ns	ns	ns	ns	ns	ns	ns	ns	ns	ns	ns	ns	ns	ns	ns	ns	ns	ns	ns	ns	ns	**	ns	ns	*	ns	****	ns	ns
Ovis_aries										ns	****	ns	ns	*	ns	ns	ns	ns	ns	ns	ns	ns	**	**	**	ns	ns	*	ns	ns	ns	ns	ns	ns	ns	ns	ns	ns	ns	ns
Orcinus_orca											ns	**	ns	****	***	****	****	****	***	****	****	****	****	****	****	ns	***	****	****	****	ns	*	ns	ns	ns	ns	ns	ns	ns	****
Tursiops_truncatus												****	****	****	****	****	****	****	****	****	****	****	****	****	****	****	****	****	****	****	****	****	**	****	****	**	***	ns	***	****
Felis_catus												ns	ns	ns	ns	ns	ns	ns	ns	ns	ns	ns	ns	ns	ns	ns	ns	ns	ns	ns	ns	ns	ns	ns	ns	ns	ns	*	ns	ns
Ailuropoda_melanoleuca													ns	ns	ns	ns	ns	ns	ns	ns	ns	ns	ns	ns	ns	ns	ns	ns	ns	ns	ns	ns	ns	ns	ns	ns	ns	ns	ns	ns
Mustela_putorius_furo														ns	ns	ns	ns	ns	ns	ns	ns	ns	ns	ns	ns	ns	ns	ns	ns	ns	*	ns	****	*	ns	***	**	****	**	ns
Odobenus_rosmarus_divergens															ns	ns	ns	ns	ns	ns	ns	ns	ns	ns	ns	ns	ns	ns	ns	ns	ns	ns	*	ns	ns	ns	ns	***	ns	ns
Canis_lupus_familiaris																ns	ns	ns	ns	ns	ns	ns	ns	ns	ns	ns	ns	ns	ns	ns	ns	ns	**	ns	ns	**	ns	****	ns	ns
Heterocephalus_glaber																	ns	ns	ns	ns	ns	ns	ns	ns	ns	ns	ns	ns	ns	ns	ns	ns	**	ns	ns	**	*	****	*	ns
Octodon_degus																		ns	ns	ns	ns	ns	ns	ns	ns	ns	ns	ns	ns	ns	*	ns	***	ns	ns	***	**	****	**	ns
Cavia_porcellus																			ns	ns	ns	ns	ns	ns	ns	ns	ns	ns	ns	ns	ns	ns	ns	ns	ns	ns	ns	***	ns	ns
Chinchilla_lanigera																				ns	ns	ns	ns	ns	ns	ns	ns	ns	ns	ns	ns	ns	***	ns	ns	**	*	****	*	ns
Ictidomys_tridecemlineatus																					ns	ns	ns	ns	ns	ns	ns	ns	ns	ns	ns	ns	**	ns	ns	**	ns	****	*	ns
Jaculus_jaculus																						ns	ns	ns	ns	ns	ns	ns	ns	ns	ns	ns	***	ns	ns	***	*	****	**	ns
Mus_musculus																							ns	ns	ns	ns	ns	ns	ns	ns	***	ns	****	**	*	****	****	****	****	ns
Rattus_norvegicus																								ns	ns	ns	ns	ns	ns	ns	**	ns	****	**	ns	****	***	****	***	ns
Microtus_ochrogaster																									ns	ns	ns	ns	ns	ns	**	ns	****	**	ns	****	***	****	***	ns
Cricetulus_griseus																										ns	ns	ns	ns	ns	ns	ns	ns	ns	ns	ns	ns	ns	ns	ns
Mesocricetus_auratus																											ns	ns	ns	ns	ns	ns	*	ns	ns	ns	ns	***	ns	ns
Ochotona_princeps																												ns	ns	ns	**	ns	****	*	ns	***	**	****	**	ns
Oryctolagus_cuniculus																													ns	ns	ns	ns	**	ns	ns	*	ns	****	ns	ns
Otolemur_garnettii																														ns	ns	ns	*	ns	ns	*	ns	***	ns	ns
Callithrix_jacchus																															ns	ns	ns	ns	ns	ns	ns	ns	ns	ns
Saimiri_boliviensis																																ns	ns	ns	ns	ns	ns	*	ns	ns
Macaca_mulatta																																	ns	ns	ns	ns	ns	ns	ns	***
Papio_anubis																																		ns	ns	ns	ns	ns	ns	ns
Nomascus_leucogenys																																			ns	ns	ns	ns	ns	ns
Pongo_abelii																																				ns	ns	ns	ns	***
Gorilla_gorilla																																					ns	ns	ns	*
Pan_paniscus																																						ns	ns	****
Pan_troglodytes																																								**

Figure 3F. Genetic Association Database - Eating Disorders

	Loxodonta_africana	Trichechus_manatus_latirostris	Sorex_araneus	Condylura_cristata	Equus_caballus	Ceratotherium_simum_simum	Sus_scrofa	Bos_taurus	Ovis_aries	Orcinus_orca	Tursiops_truncatus	Felis_catus	Ailuropoda_melanoleuca	Mustela_putorius_furo	Odobenus_rosmarus_divergens	Canis_lupus_familiaris	Heterocephalus_glaber	Octodon_degus	Cavia_porcellus	Chinchilla_lanigera	Ictidomys_tridecemlineatus	Jaculus_jaculus	Mus_musculus	Rattus_norvegicus	Microtus_ochrogaster	Cricetulus_griseus	Mesocricetus_auratus	Ochotona_princeps	Oryctolagus_cuniculus	Otolemur_garnettii	Callithrix_jacchus	Saimiri_boliviensis	Macaca_mulatta	Papio_anubis	Nomascus_leucogenys	Pongo_abelii	Gorilla_gorilla	Pan_paniscus	Pan_troglodytes	Homo_sapiens
Echinops_telfairi	ns	ns	ns	ns	ns	ns	ns	ns	ns	ns	ns	ns	ns	ns	ns	ns	ns	ns	ns	ns	ns	ns	ns	ns	ns	ns	ns	ns	ns	ns	ns	ns	ns	ns	ns	ns	ns	ns	ns	ns
Loxodonta_africana		ns	ns	ns	ns	ns	ns	ns	ns	ns	ns	ns	ns	ns	ns	ns	ns	ns	ns	ns	ns	ns	ns	ns	ns	ns	ns	ns	ns	ns	ns	ns	ns	ns	ns	ns	ns	ns	ns	ns
Trichechus_manatus_latirostris			ns	ns	ns	ns	ns	ns	ns	ns	ns	ns	ns	ns	ns	ns	ns	ns	ns	ns	ns	ns	ns	ns	ns	ns	ns	ns	ns	ns	ns	ns	ns	ns	ns	ns	ns	ns	ns	ns
Sorex_araneus				ns	ns	ns	ns	ns	ns	ns	ns	ns	ns	ns	ns	ns	ns	ns	ns	ns	ns	ns	ns	ns	ns	ns	ns	ns	ns	ns	ns	ns	ns	ns	ns	ns	ns	ns	ns	ns
Condylura_cristata					ns	ns	ns	ns	ns	ns	ns	ns	ns	ns	ns	ns	ns	ns	ns	ns	ns	ns	ns	ns	ns	ns	ns	ns	ns	ns	ns	ns	ns	ns	ns	ns	ns	ns	ns	ns
Equus_caballus						ns	ns	ns	ns	ns	ns	ns	ns	ns	ns	ns	ns	ns	ns	ns	ns	ns	ns	ns	ns	ns	ns	ns	ns	ns	ns	ns	ns	ns	ns	ns	ns	ns	ns	ns
Ceratotherium_simum_simum							ns	ns	ns	ns	ns	ns	ns	ns	ns	ns	ns	ns	ns	ns	ns	ns	ns	ns	ns	ns	ns	ns	ns	ns	ns	ns	ns	ns	ns	ns	ns	ns	ns	ns
Sus_scrofa								ns	ns	ns	ns	ns	ns	ns	ns	ns	ns	ns	ns	ns	ns	ns	ns	ns	ns	ns	ns	ns	ns	ns	ns	ns	ns	ns	ns	ns	ns	ns	ns	ns
Bos_taurus									ns	ns	ns	ns	ns	ns	ns	ns	ns	ns	ns	ns	ns	ns	ns	ns	ns	ns	ns	ns	ns	ns	ns	ns	ns	ns	ns	ns	ns	ns	ns	ns
Ovis_aries										ns	ns	ns	ns	ns	ns	ns	ns	ns	ns	ns	ns	ns	ns	ns	ns	ns	ns	ns	ns	ns	ns	ns	ns	ns	ns	ns	ns	ns	ns	ns
Orcinus_orca											ns	ns	ns	ns	ns	ns	ns	ns	ns	ns	ns	ns	ns	ns	ns	ns	ns	ns	ns	ns	ns	ns	ns	ns	ns	ns	ns	ns	ns	ns
Tursiops_truncatus												ns	ns	ns	ns	ns	ns	ns	ns	ns	ns	ns	ns	ns	ns	ns	ns	ns	ns	ns	ns	ns	ns	ns	ns	ns	ns	ns	ns	ns
Felis_catus													ns	ns	ns	ns	ns	ns	ns	ns	ns	ns	ns	ns	ns	ns	ns	ns	ns	ns	ns	ns	ns	ns	ns	ns	ns	ns	ns	ns
Ailuropoda_melanoleuca														ns	ns	ns	ns	ns	ns	ns	ns	ns	ns	ns	ns	ns	ns	ns	ns	ns	ns	ns	ns	ns	ns	ns	ns	ns	ns	ns
Mustela_putorius_furo															ns	ns	ns	ns	ns	ns	ns	ns	ns	ns	ns	ns	ns	ns	ns	ns	ns	ns	ns	ns	ns	ns	ns	ns	ns	ns
Odobenus_rosmarus_divergens																ns	ns	ns	ns	ns	ns	ns	ns	ns	ns	ns	ns	ns	ns	ns	ns	ns	ns	ns	ns	ns	ns	ns	ns	ns
Canis_lupus_familiaris																	ns	ns	ns	ns	ns	ns	ns	ns	ns	ns	ns	ns	ns	ns	ns	ns	ns	ns	ns	ns	ns	ns	ns	ns
Heterocephalus_glaber																		ns	ns	ns	ns	ns	ns	ns	ns	ns	ns	ns	ns	ns	ns	ns	ns	ns	ns	ns	ns	ns	ns	ns
Octodon_degus																			ns	ns	ns	ns	ns	ns	ns	ns	ns	ns	ns	ns	ns	ns	ns	ns	ns	ns	ns	ns	ns	ns
Cavia_porcellus																				ns	ns	ns	ns	ns	ns	ns	ns	ns	ns	ns	ns	ns	ns	ns	ns	ns	ns	ns	ns	ns
Chinchilla_lanigera																					ns	ns	ns	ns	ns	ns	ns	ns	ns	ns	ns	ns	ns	ns	ns	ns	ns	ns	ns	ns
Ictidomys_tridecemlineatus																						ns	ns	ns	ns	ns	ns	ns	ns	ns	ns	ns	ns	ns	ns	ns	ns	ns	ns	ns
Jaculus_jaculus																							ns	ns	ns	ns	ns	ns	ns	ns	ns	ns	ns	ns	ns	ns	ns	ns	ns	ns
Mus_musculus																								ns	ns	ns	ns	ns	ns	ns	ns	ns	ns	ns	ns	ns	ns	ns	ns	ns
Rattus_norvegicus																									ns	ns	ns	ns	ns	ns	ns	ns	ns	ns	ns	ns	ns	ns	ns	ns
Microtus_ochrogaster																										ns	ns	ns	ns	ns	ns	ns	ns	ns	ns	ns	ns	ns	ns	ns
Cricetulus_griseus																											ns	ns	ns	ns	ns	ns	ns	ns	ns	ns	ns	ns	ns	ns
Mesocricetus_auratus																												ns	ns	ns	ns	ns	ns	ns	ns	ns	ns	ns	ns	ns
Ochotona_princeps																													ns	ns	ns	ns	ns	ns	ns	ns	ns	ns	ns	ns
Oryctolagus_cuniculus																														ns	ns	ns	ns	ns	ns	ns	ns	ns	ns	ns
Otolemur_garnettii																															ns	ns	ns	ns	ns	ns	ns	ns	ns	ns
Callithrix_jacchus																																ns	ns	ns	ns	ns	ns	ns	ns	ns
Saimiri_boliviensis																																	ns	ns	ns	ns	ns	ns	ns	ns
Macaca_mulatta																																		ns	ns	ns	ns	ns	ns	ns
Papio_anubis																																			ns	ns	ns	ns	ns	ns
Nomascus_leucogenys																																				ns	ns	ns	ns	ns
Pongo_abelii																																					ns	ns	ns	ns
Gorilla_gorilla																																						ns	ns	ns
Pan_paniscus																																							ns	ns
Pan_troglodytes																																								ns

Figure 3G. Genetic Association Database - Alzheimers

	Loxodonta_africana	Trichechus_manatus_latirostris	Sorex_araneus	Condylura_cristata	Equus_caballus	Ceratotherium_simum_simum	Sus_scrofa	Bos_taurus	Ovis_aries	Orcinus_orca	Tursiops_truncatus	Felis_catus	Ailuropoda_melanoleuca	Mustela_putorius_furo	Odobenus_rosmarus_divergens	Canis_lupus_familiaris	Heterocephalus_glaber	Octodon_degus	Cavia_porcellus	Chinchilla_lanigera	Ictidomys_tridecemlineatus	Jaculus_jaculus	Mus_musculus	Rattus_norvegicus	Microtus_ochrogaster	Cricetulus_griseus	Mesocricetus_auratus	Ochotona_princeps	Oryctolagus_cuniculus	Otolemur_garnettii	Callithrix_jacchus	Saimiri_boliviensis	Macaca_mulatta	Papio_anubis	Nomascus_leucogenys	Pongo_abelii	Gorilla_gorilla	Pan_paniscus	Pan_troglodytes	Homo_sapiens
Echinops_telfairi	ns	ns	ns	ns	ns	ns	ns	ns	ns	ns	****	ns	ns	ns	ns	ns	ns	ns	ns	ns	ns	ns	ns	ns	ns	ns	ns	ns	ns	ns	ns	ns	****	ns	ns	ns	*	ns	ns	ns
Loxodonta_africana		ns	ns	ns	ns	ns	ns	ns	ns	ns	****	ns	ns	ns	ns	ns	ns	ns	ns	ns	ns	ns	*	ns	ns	ns	ns	ns	ns	ns	ns	ns	ns	ns	ns	ns	ns	ns	ns	ns
Trichechus_manatus_latirostris			ns	ns	ns	ns	ns	ns	ns	ns	****	ns	ns	ns	ns	ns	ns	ns	ns	ns	ns	ns	ns	ns	ns	ns	ns	ns	ns	ns	ns	ns	ns	ns	ns	ns	ns	ns	ns	ns
Sorex_araneus				ns	ns	ns	ns	ns	ns	ns	****	ns	ns	ns	ns	ns	ns	ns	ns	ns	ns	ns	ns	ns	ns	ns	ns	ns	ns	ns	ns	ns	****	*	ns	*	***	ns	ns	ns
Condylura_cristata					ns	ns	ns	ns	ns	ns	****	ns	ns	ns	ns	ns	ns	ns	ns	ns	ns	ns	ns	ns	ns	ns	ns	ns	ns	ns	ns	ns	****	ns	ns	ns	**	ns	ns	ns
Equus_caballus						ns	ns	ns	ns	ns	****	ns	ns	ns	ns	ns	ns	ns	ns	ns	ns	ns	ns	ns	ns	ns	ns	ns	ns	ns	ns	ns	*	ns	ns	ns	ns	ns	ns	ns
Ceratotherium_simum_simum							ns	ns	ns	ns	****	ns	ns	ns	ns	ns	ns	ns	ns	ns	ns	ns	ns	ns	ns	ns	ns	ns	ns	ns	ns	ns	**	ns	ns	ns	ns	ns	ns	ns
Sus_scrofa								ns	ns	ns	****	ns	ns	ns	ns	ns	ns	ns	ns	ns	ns	ns	ns	ns	ns	ns	ns	ns	ns	ns	ns	ns	**	ns	ns	ns	ns	ns	ns	ns
Bos_taurus									ns	ns	****	ns	ns	ns	ns	ns	ns	ns	ns	ns	ns	ns	ns	ns	ns	ns	ns	ns	ns	ns	ns	ns	****	ns	ns	ns	**	ns	ns	ns
Ovis_aries										ns	****	ns	ns	ns	ns	ns	ns	ns	ns	ns	ns	ns	*	ns	ns	ns	ns	ns	ns	ns	ns	ns	ns	ns	ns	ns	ns	ns	ns	ns
Orcinus_orca											****	ns	ns	ns	ns	ns	ns	ns	ns	ns	ns	ns	ns	ns	ns	ns	ns	ns	ns	ns	ns	ns	ns	ns	ns	ns	ns	ns	ns	ns
Tursiops_truncatus												****	****	****	****	****	****	****	****	****	****	****	****	****	****	****	****	****	****	****	****	****	ns	***	****	***	*	****	****	****
Felis_catus													ns	ns	ns	ns	ns	ns	ns	ns	ns	ns	ns	ns	ns	ns	ns	ns	ns	ns	ns	ns	***	ns	ns	ns	ns	ns	ns	ns
Ailuropoda_melanoleuca														ns	ns	ns	ns	ns	ns	ns	ns	ns	ns	ns	ns	ns	ns	ns	ns	ns	ns	ns	*	ns	ns	ns	ns	ns	ns	ns
Mustela_putorius_furo															ns	ns	ns	ns	ns	ns	ns	ns	ns	ns	ns	ns	ns	ns	ns	ns	ns	ns	****	*	ns	*	***	ns	ns	ns
Odobenus_rosmarus_divergens																ns	ns	ns	ns	ns	ns	ns	ns	ns	ns	ns	ns	ns	ns	ns	ns	ns	****	ns	ns	ns	*	ns	ns	ns
Canis_lupus_familiaris																	ns	ns	ns	ns	ns	ns	ns	ns	ns	ns	ns	ns	ns	ns	ns	ns	****	ns	ns	ns	ns	ns	ns	ns
Heterocephalus_glaber																		ns	ns	ns	ns	ns	ns	ns	ns	ns	ns	ns	ns	ns	ns	ns	****	ns	ns	ns	ns	ns	ns	ns
Octodon_degus																			ns	ns	ns	ns	ns	ns	ns	ns	ns	ns	ns	ns	ns	ns	****	ns	ns	ns	*	ns	ns	ns
Cavia_porcellus																				ns	ns	ns	ns	ns	ns	ns	ns	ns	ns	ns	ns	ns	***	ns	ns	ns	ns	ns	ns	ns
Chinchilla_lanigera																					ns	ns	ns	ns	ns	ns	ns	ns	ns	ns	ns	ns	****	ns	ns	ns	**	ns	ns	ns
Ictidomys_tridecemlineatus																						ns	ns	ns	ns	ns	ns	ns	ns	ns	ns	ns	****	ns	ns	ns	**	ns	ns	ns
Jaculus_jaculus																							ns	ns	ns	ns	ns	ns	ns	ns	ns	ns	****	ns	ns	ns	***	ns	ns	ns
Mus_musculus																								ns	ns	ns	ns	ns	ns	ns	ns	ns	****	**	ns	**	****	*	ns	ns
Rattus_norvegicus																									ns	ns	ns	ns	ns	ns	ns	ns	****	*	ns	*	***	ns	ns	ns
Microtus_ochrogaster																										ns	ns	ns	ns	ns	ns	ns	****	ns	ns	ns	**	ns	ns	ns
Cricetulus_griseus																											ns	ns	ns	ns	ns	ns	ns	ns	ns	ns	ns	ns	ns	ns
Mesocricetus_auratus																												ns	ns	ns	ns	ns	***	ns	ns	ns	ns	ns	ns	ns
Ochotona_princeps																													ns	ns	ns	ns	****	ns	ns	ns	**	ns	ns	ns
Oryctolagus_cuniculus																														ns	ns	ns	****	ns	ns	ns	*	ns	ns	ns
Otolemur_garnettii																															ns	ns	***	ns	ns	ns	ns	ns	ns	ns
Callithrix_jacchus																																ns	ns	ns	ns	ns	ns	ns	ns	ns
Saimiri_boliviensis																																	***	ns	ns	ns	ns	ns	ns	ns
Macaca_mulatta																																		ns	ns	ns	ns	ns	ns	***
Papio_anubis																																			ns	ns	ns	ns	ns	ns
Nomascus_leucogenys																																				ns	ns	ns	ns	ns
Pongo_abelii																																					ns	ns	ns	ns
Gorilla_gorilla																																						ns	ns	ns
Pan_paniscus																																							ns	ns
Pan_troglodytes																																								ns

Figure 3H. Genetic Association Database - Parkinsons

	Loxodonta_africana	Trichechus_manatus_latirostris	Sorex_araneus	Condylura_cristata	Equus_caballus	Ceratotherium_simum_simum	Sus_scrofa	Bos_taurus	Ovis_aries	Orcinus_orca	Tursiops_truncatus	Felis_catus	Ailuropoda_melanoleuca	Mustela_putorius_furo	Odobenus_rosmarus_divergens	Canis_lupus_familiaris	Heterocephalus_glaber	Octodon_degus	Cavia_porcellus	Chinchilla_lanigera	Ictidomys_tridecemlineatus	Jaculus_jaculus	Mus_musculus	Rattus_norvegicus	Microtus_ochrogaster	Cricetulus_griseus	Mesocricetus_auratus	Ochotona_princeps	Oryctolagus_cuniculus	Otolemur_garnettii	Callithrix_jacchus	Saimiri_boliviensis	Macaca_mulatta	Papio_anubis	Nomascus_leucogenys	Pongo_abelii	Gorilla_gorilla	Pan_paniscus	Pan_troglodytes	Homo_sapiens
Echinops_telfairi	ns	ns	ns	ns	ns	ns	ns	ns	ns	ns	****	ns	ns	ns	ns	ns	ns	ns	ns	ns	ns	ns	ns	ns	ns	ns	ns	ns	ns	ns	ns	ns	****	ns	ns	ns	ns	ns	****	ns
Loxodonta_africana		ns	ns	ns	ns	ns	ns	ns	ns	ns	*	ns	ns	ns	ns	ns	ns	ns	ns	ns	ns	ns	ns	ns	ns	ns	ns	ns	ns	ns	ns	ns	ns	ns	ns	ns	ns	ns	*	ns
Trichechus_manatus_latirostris			ns	ns	ns	ns	ns	ns	ns	ns	***	ns	ns	ns	ns	ns	ns	ns	ns	ns	ns	ns	ns	ns	ns	ns	ns	ns	ns	ns	ns	ns	**	ns	ns	ns	ns	ns	***	ns
Sorex_araneus				ns	ns	ns	ns	ns	ns	ns	****	ns	ns	ns	ns	ns	ns	ns	ns	ns	ns	ns	ns	ns	ns	ns	ns	ns	ns	ns	ns	ns	****	*	ns	ns	ns	ns	****	ns
Condylura_cristata					ns	ns	ns	ns	ns	ns	****	ns	ns	ns	ns	ns	ns	ns	ns	ns	ns	ns	ns	ns	ns	ns	ns	ns	ns	ns	ns	ns	***	ns	ns	ns	ns	ns	****	ns
Equus_caballus						ns	ns	ns	ns	ns	**	ns	ns	ns	ns	ns	ns	ns	ns	ns	ns	ns	ns	ns	ns	ns	ns	ns	ns	ns	ns	ns	ns	ns	ns	ns	ns	ns	**	ns
Ceratotherium_simum_simum							ns	ns	ns	ns	****	ns	ns	ns	ns	ns	ns	ns	ns	ns	ns	ns	ns	ns	ns	ns	ns	ns	ns	ns	ns	ns	***	ns	ns	ns	ns	ns	****	ns
Sus_scrofa								ns	ns	ns	***	ns	ns	ns	ns	ns	ns	ns	ns	ns	ns	ns	ns	ns	ns	ns	ns	ns	ns	ns	ns	ns	**	ns	ns	ns	ns	ns	****	ns
Bos_taurus									ns	ns	****	ns	ns	ns	ns	ns	ns	ns	ns	ns	ns	ns	ns	ns	ns	ns	ns	ns	ns	ns	ns	ns	***	ns	ns	ns	ns	ns	****	ns
Ovis_aries										ns	*	ns	ns	ns	ns	ns	ns	ns	ns	ns	ns	ns	ns	ns	ns	ns	ns	ns	ns	ns	ns	ns	ns	ns	ns	ns	ns	ns	*	ns
Orcinus_orca											ns	ns	ns	ns	ns	ns	ns	ns	ns	ns	ns	ns	ns	ns	ns	ns	ns	ns	ns	ns	ns	ns	ns	ns	ns	ns	ns	ns	ns	ns
Tursiops_truncatus												****	***	****	****	****	****	****	****	****	****	****	****	****	****	*	***	****	****	****	ns	*	ns	ns	ns	ns	ns	ns	ns	*
Felis_catus													ns	ns	ns	ns	ns	ns	ns	ns	ns	ns	ns	ns	ns	ns	ns	ns	ns	ns	ns	ns	**	ns	ns	ns	ns	ns	****	ns
Ailuropoda_melanoleuca														ns	ns	ns	ns	ns	ns	ns	ns	ns	ns	ns	ns	ns	ns	ns	ns	ns	ns	ns	*	ns	ns	ns	ns	ns	***	ns
Mustela_putorius_furo															ns	ns	ns	ns	ns	ns	ns	ns	ns	ns	ns	ns	ns	ns	ns	ns	ns	ns	****	*	ns	ns	ns	ns	****	ns
Odobenus_rosmarus_divergens																ns	ns	ns	ns	ns	ns	ns	ns	ns	ns	ns	ns	ns	ns	ns	ns	ns	****	ns	ns	ns	ns	ns	****	ns
Canis_lupus_familiaris																	ns	ns	ns	ns	ns	ns	ns	ns	ns	ns	ns	ns	ns	ns	ns	ns	****	ns	ns	ns	ns	ns	****	ns
Heterocephalus_glaber																		ns	ns	ns	ns	ns	ns	ns	ns	ns	ns	ns	ns	ns	ns	ns	***	ns	ns	ns	ns	ns	****	ns
Octodon_degus																			ns	ns	ns	ns	ns	ns	ns	ns	ns	ns	ns	ns	ns	ns	****	ns	ns	ns	ns	ns	****	ns
Cavia_porcellus																				ns	ns	ns	ns	ns	ns	ns	ns	ns	ns	ns	ns	ns	***	ns	ns	ns	ns	ns	****	ns
Chinchilla_lanigera																					ns	ns	ns	ns	ns	ns	ns	ns	ns	ns	ns	ns	***	ns	ns	ns	ns	ns	****	ns
Ictidomys_tridecemlineatus																						ns	ns	ns	ns	ns	ns	ns	ns	ns	ns	ns	***	ns	ns	ns	ns	ns	****	ns
Jaculus_jaculus																							ns	ns	ns	ns	ns	ns	ns	ns	ns	ns	****	ns	ns	ns	ns	ns	****	ns
Mus_musculus																								ns	ns	ns	ns	ns	ns	ns	*	ns	****	*	ns	ns	ns	ns	****	ns
Rattus_norvegicus																									ns	ns	ns	ns	ns	ns	*	ns	****	**	ns	ns	ns	ns	****	ns
Microtus_ochrogaster																										ns	ns	ns	ns	ns	*	ns	****	**	ns	ns	ns	*	****	ns
Cricetulus_griseus																											ns	ns	ns	ns	ns	ns	ns	ns	ns	ns	ns	ns	*	ns
Mesocricetus_auratus																												ns	ns	ns	ns	ns	**	ns	ns	ns	ns	ns	****	ns
Ochotona_princeps																													ns	ns	ns	ns	****	ns	ns	ns	ns	ns	****	ns
Oryctolagus_cuniculus																														ns	ns	ns	***	ns	ns	ns	ns	ns	****	ns
Otolemur_garnettii																															ns	ns	****	ns	ns	ns	ns	ns	****	ns
Callithrix_jacchus																																ns	ns	ns	ns	ns	ns	ns	ns	ns
Saimiri_boliviensis																																	ns	ns	ns	ns	ns	ns	*	ns
Macaca_mulatta																																		ns	ns	ns	ns	ns	ns	ns
Papio_anubis																																			ns	ns	ns	ns	ns	ns
Nomascus_leucogenys																																				ns	ns	ns	ns	ns
Pongo_abelii																																					ns	ns	ns	ns
Gorilla_gorilla																																						ns	ns	ns
Pan_paniscus																																							ns	ns
Pan_troglodytes																																								**

Figure 3H. Genetic Association Database - Migraine

	Loxodonta_africana	Trichechus_manatus_latirostris	Sorex_araneus	Condylura_cristata	Equus_caballus	Ceratotherium_simum_simum	Sus_scrofa	Bos_taurus	Ovis_aries	Orcinus_orca	Tursiops_truncatus	Felis_catus	Ailuropoda_melanoleuca	Mustela_putorius_furo	Odobenus_rosmarus_divergens	Canis_lupus_familiaris	Heterocephalus_glaber	Octodon_degus	Cavia_porcellus	Chinchilla_lanigera	Ictidomys_tridecemlineatus	Jaculus_jaculus	Mus_musculus	Rattus_norvegicus	Microtus_ochrogaster	Cricetulus_griseus	Mesocricetus_auratus	Ochotona_princeps	Oryctolagus_cuniculus	Otolemur_garnettii	Callithrix_jacchus	Saimiri_boliviensis	Macaca_mulatta	Papio_anubis	Nomascus_leucogenys	Pongo_abelii	Gorilla_gorilla	Pan_paniscus	Pan_troglodytes	Homo_sapiens
Echinops_telfairi	ns	ns	ns	ns	ns	ns	ns	ns	ns	ns	ns	ns	ns	ns	ns	ns	ns	ns	ns	ns	ns	ns	ns	ns	ns	ns	ns	ns	ns	ns	ns	ns	ns	ns	ns	ns	ns	ns	ns	ns
Loxodonta_africana		ns	ns	ns	ns	ns	ns	ns	ns	ns	ns	ns	ns	ns	ns	ns	ns	ns	ns	ns	ns	ns	ns	ns	ns	ns	ns	ns	ns	ns	ns	ns	ns	ns	ns	ns	ns	ns	ns	ns
Trichechus_manatus_latirostris			ns	ns	ns	ns	ns	ns	ns	ns	ns	ns	ns	ns	ns	ns	ns	ns	ns	ns	ns	ns	ns	ns	ns	ns	ns	ns	ns	ns	ns	ns	ns	ns	ns	ns	ns	ns	ns	ns
Sorex_araneus				ns	ns	ns	ns	ns	ns	ns	ns	ns	ns	ns	ns	ns	ns	ns	ns	ns	ns	ns	ns	ns	ns	ns	ns	ns	ns	ns	ns	ns	ns	ns	ns	ns	ns	ns	ns	ns
Condylura_cristata					ns	ns	ns	ns	ns	ns	ns	ns	ns	ns	ns	ns	ns	ns	ns	ns	ns	ns	ns	ns	ns	ns	ns	ns	ns	ns	ns	ns	ns	ns	ns	ns	ns	ns	ns	ns
Equus_caballus						ns	ns	ns	ns	ns	ns	ns	ns	ns	ns	ns	ns	ns	ns	ns	ns	ns	ns	ns	ns	ns	ns	ns	ns	ns	ns	ns	ns	ns	ns	ns	ns	ns	ns	ns
Ceratotherium_simum_simum							ns	ns	ns	ns	ns	ns	ns	ns	ns	ns	ns	ns	ns	ns	ns	ns	ns	ns	ns	ns	ns	ns	ns	ns	ns	ns	ns	ns	ns	ns	ns	ns	ns	ns
Sus_scrofa								ns	ns	ns	ns	ns	ns	ns	ns	ns	ns	ns	ns	ns	ns	ns	ns	ns	ns	ns	ns	ns	ns	ns	ns	ns	ns	ns	ns	ns	ns	ns	ns	ns
Bos_taurus									ns	ns	ns	ns	ns	ns	ns	ns	ns	ns	ns	ns	ns	ns	ns	ns	ns	ns	ns	ns	ns	ns	ns	ns	ns	ns	ns	ns	ns	ns	ns	ns
Ovis_aries										ns	ns	ns	ns	ns	ns	ns	ns	ns	ns	ns	ns	ns	ns	ns	ns	ns	ns	ns	ns	ns	ns	ns	ns	ns	ns	ns	ns	ns	ns	ns
Orcinus_orca											ns	ns	ns	ns	ns	ns	ns	ns	ns	ns	ns	ns	ns	ns	ns	ns	ns	ns	ns	ns	ns	ns	ns	ns	ns	ns	ns	ns	ns	ns
Tursiops_truncatus												ns	ns	ns	ns	ns	ns	ns	ns	ns	ns	ns	ns	*	ns	ns	ns	ns	ns	ns	ns	ns	ns	ns	ns	ns	ns	ns	ns	ns
Felis_catus													ns	ns	ns	ns	ns	ns	ns	ns	ns	ns	ns	ns	ns	ns	ns	ns	ns	ns	ns	ns	ns	ns	ns	ns	ns	ns	ns	ns
Ailuropoda_melanoleuca														ns	ns	ns	ns	ns	ns	ns	ns	ns	ns	ns	ns	ns	ns	ns	ns	ns	ns	ns	ns	ns	ns	ns	ns	ns	ns	ns
Mustela_putorius_furo															ns	ns	ns	ns	ns	ns	ns	ns	ns	ns	ns	ns	ns	ns	ns	ns	ns	ns	ns	ns	ns	ns	ns	ns	ns	ns
Odobenus_rosmarus_divergens																ns	ns	ns	ns	ns	ns	ns	ns	ns	ns	ns	ns	ns	ns	ns	ns	ns	ns	ns	ns	ns	ns	ns	ns	ns
Canis_lupus_familiaris																	ns	ns	ns	ns	ns	ns	ns	ns	ns	ns	ns	ns	ns	ns	ns	ns	ns	ns	ns	ns	ns	ns	ns	ns
Heterocephalus_glaber																		ns	ns	ns	ns	ns	ns	ns	ns	ns	ns	ns	ns	ns	ns	ns	ns	ns	ns	ns	ns	ns	ns	ns
Octodon_degus																			ns	ns	ns	ns	ns	ns	ns	ns	ns	ns	ns	ns	ns	ns	ns	ns	ns	ns	ns	ns	ns	ns
Cavia_porcellus																				ns	ns	ns	ns	ns	ns	ns	ns	ns	ns	ns	ns	ns	ns	ns	ns	ns	ns	ns	ns	ns
Chinchilla_lanigera																					ns	ns	ns	ns	ns	ns	ns	ns	ns	ns	ns	ns	ns	ns	ns	ns	ns	ns	ns	ns
Ictidomys_tridecemlineatus																						ns	ns	ns	ns	ns	ns	ns	ns	ns	ns	ns	ns	ns	ns	ns	ns	ns	ns	ns
Jaculus_jaculus																							ns	ns	ns	ns	ns	ns	ns	ns	ns	ns	ns	ns	ns	ns	ns	ns	ns	ns
Mus_musculus																								ns	ns	ns	ns	ns	ns	ns	ns	ns	ns	ns	ns	ns	ns	ns	ns	ns
Rattus_norvegicus																									ns	ns	ns	ns	ns	ns	ns	ns	ns	ns	ns	ns	ns	ns	ns	ns
Microtus_ochrogaster																										ns	ns	ns	ns	ns	ns	ns	ns	ns	ns	ns	ns	ns	ns	ns
Cricetulus_griseus																											ns	ns	ns	ns	ns	ns	ns	ns	ns	ns	ns	ns	ns	ns
Mesocricetus_auratus																												ns	ns	ns	ns	ns	ns	ns	ns	ns	ns	ns	ns	ns
Ochotona_princeps																													ns	ns	ns	ns	ns	ns	ns	ns	ns	ns	ns	ns
Oryctolagus_cuniculus																														ns	ns	ns	ns	ns	ns	ns	ns	ns	ns	ns
Otolemur_garnettii																															ns	ns	ns	ns	ns	ns	ns	ns	ns	ns
Callithrix_jacchus																																ns	ns	ns	ns	ns	ns	ns	ns	ns
Saimiri_boliviensis																																	ns	ns	ns	ns	ns	ns	ns	ns
Macaca_mulatta																																		ns	ns	ns	ns	ns	ns	ns
Papio_anubis																																			ns	ns	ns	ns	ns	ns
Nomascus_leucogenys																																				ns	ns	ns	ns	ns
Pongo_abelii																																					ns	ns	ns	ns
Gorilla_gorilla																																						ns	ns	ns
Pan_paniscus																																							ns	ns
Pan_troglodytes																																								ns

Figure 3I. Genetic Association Database - Stroke

	Loxodonta_africana	Trichechus_manatus_latirostris	Sorex_araneus	Condylura_cristata	Equus_caballus	Ceratotherium_simum_simum	Sus_scrofa	Bos_taurus	Ovis_aries	Orcinus_orca	Tursiops_truncatus	Felis_catus	Ailuropoda_melanoleuca	Mustela_putorius_furo	Odobenus_rosmarus_divergens	Canis_lupus_familiaris	Heterocephalus_glaber	Octodon_degus	Cavia_porcellus	Chinchilla_lanigera	Ictidomys_tridecemlineatus	Jaculus_jaculus	Mus_musculus	Rattus_norvegicus	Microtus_ochrogaster	Cricetulus_griseus	Mesocricetus_auratus	Ochotona_princeps	Oryctolagus_cuniculus	Otolemur_garnettii	Callithrix_jacchus	Saimiri_boliviensis	Macaca_mulatta	Papio_anubis	Nomascus_leucogenys	Pongo_abelii	Gorilla_gorilla	Pan_paniscus	Pan_troglodytes	Homo_sapiens
Echinops_telfairi	ns	ns	ns	ns	ns	ns	ns	ns	ns	*	****	ns	ns	ns	ns	ns	ns	ns	ns	ns	ns	ns	ns	ns	ns	ns	ns	ns	ns	ns	ns	ns	ns	****	ns	**	**	ns	****	ns
Loxodonta_africana		ns	ns	ns	ns	ns	ns	ns	ns	ns	****	ns	ns	ns	ns	ns	ns	ns	ns	ns	ns	ns	**	**	**	ns	ns	ns	ns	ns	ns	ns	ns	ns	ns	ns	ns	ns	*	ns
Trichechus_manatus_latirostris			ns	ns	ns	ns	ns	ns	ns	ns	****	ns	ns	ns	ns	ns	ns	ns	ns	ns	ns	ns	ns	ns	ns	ns	ns	ns	ns	ns	ns	ns	ns	ns	ns	ns	ns	ns	***	ns
Sorex_araneus				ns	ns	ns	ns	ns	ns	*	****	ns	ns	ns	ns	ns	ns	ns	ns	ns	ns	ns	ns	ns	ns	ns	ns	ns	ns	ns	ns	ns	ns	****	ns	*	**	ns	****	ns
Condylura_cristata					ns	ns	ns	ns	**	***	****	ns	ns	ns	ns	ns	ns	ns	ns	ns	ns	ns	ns	ns	ns	ns	ns	ns	ns	ns	ns	ns	**	****	ns	***	****	ns	****	ns
Equus_caballus						ns	ns	ns	ns	ns	****	ns	ns	ns	ns	ns	ns	ns	ns	ns	ns	ns	ns	ns	ns	ns	ns	ns	ns	ns	ns	ns	ns	ns	ns	ns	ns	ns	***	ns
Ceratotherium_simum_simum							ns	ns	ns	*	****	ns	ns	ns	ns	ns	ns	ns	ns	ns	ns	ns	ns	ns	ns	ns	ns	ns	ns	ns	ns	ns	ns	****	ns	*	**	ns	****	ns
Sus_scrofa								ns	ns	ns	****	ns	ns	ns	ns	ns	ns	ns	ns	ns	ns	ns	ns	ns	ns	ns	ns	ns	ns	ns	ns	ns	ns	ns	ns	ns	ns	ns	***	ns
Bos_taurus									*	***	****	ns	ns	ns	ns	ns	ns	ns	ns	ns	ns	ns	ns	ns	ns	ns	ns	ns	ns	ns	ns	ns	*	****	ns	***	***	ns	****	ns
Ovis_aries										ns	****	ns	ns	*	ns	ns	ns	ns	ns	ns	ns	ns	***	***	****	ns	ns	*	ns	ns	ns	ns	ns	ns	ns	ns	ns	ns	ns	ns
Orcinus_orca											****	ns	ns	***	**	ns	*	**	ns	**	**	**	****	****	****	ns	ns	***	**	*	ns	ns	ns	ns	ns	ns	ns	ns	ns	ns
Tursiops_truncatus												****	****	****	****	****	****	****	****	****	****	****	****	****	****	****	****	****	****	****	****	****	****	****	****	****	****	****	**	****
Felis_catus													ns	ns	ns	ns	ns	ns	ns	ns	ns	ns	ns	ns	ns	ns	ns	ns	ns	ns	ns	ns	ns	**	ns	ns	ns	ns	****	ns
Ailuropoda_melanoleuca														ns	ns	ns	ns	ns	ns	ns	ns	ns	ns	ns	ns	ns	ns	ns	ns	ns	ns	ns	ns	**	ns	ns	ns	ns	****	ns
Mustela_putorius_furo															ns	ns	ns	ns	ns	ns	ns	ns	ns	ns	ns	ns	ns	ns	ns	ns	ns	ns	**	****	ns	***	***	ns	****	ns
Odobenus_rosmarus_divergens																ns	ns	ns	ns	ns	ns	ns	ns	ns	ns	ns	ns	ns	ns	ns	ns	ns	ns	****	ns	**	**	ns	****	ns
Canis_lupus_familiaris																	ns	ns	ns	ns	ns	ns	ns	ns	ns	ns	ns	ns	ns	ns	ns	ns	ns	***	ns	ns	*	ns	****	ns
Heterocephalus_glaber																		ns	ns	ns	ns	ns	ns	ns	ns	ns	ns	ns	ns	ns	ns	ns	ns	****	ns	*	**	ns	****	ns
Octodon_degus																			ns	ns	ns	ns	ns	ns	ns	ns	ns	ns	ns	ns	ns	ns	ns	****	ns	**	**	ns	****	ns
Cavia_porcellus																				ns	ns	ns	ns	ns	ns	ns	ns	ns	ns	ns	ns	ns	ns	***	ns	ns	ns	ns	****	ns
Chinchilla_lanigera																					ns	ns	ns	ns	ns	ns	ns	ns	ns	ns	ns	ns	*	****	ns	**	***	ns	****	ns
Ictidomys_tridecemlineatus																						ns	ns	ns	ns	ns	ns	ns	ns	ns	ns	ns	*	****	ns	**	**	ns	****	ns
Jaculus_jaculus																							ns	ns	ns	ns	ns	ns	ns	ns	ns	ns	ns	****	ns	**	**	ns	****	ns
Mus_musculus																								ns	ns	**	ns	ns	ns	ns	ns	ns	****	****	**	****	****	*	****	ns
Rattus_norvegicus																									ns	**	ns	ns	ns	ns	ns	ns	****	****	**	****	****	*	****	ns
Microtus_ochrogaster																										**	ns	ns	ns	ns	ns	ns	****	****	**	****	****	*	****	ns
Cricetulus_griseus																											ns	ns	ns	ns	ns	ns	ns	ns	ns	ns	ns	ns	*	ns
Mesocricetus_auratus																												ns	ns	ns	ns	ns	ns	**	ns	ns	ns	ns	****	ns
Ochotona_princeps																													ns	ns	ns	ns	**	****	ns	***	***	ns	****	ns
Oryctolagus_cuniculus																														ns	ns	ns	*	****	ns	**	***	ns	****	ns
Otolemur_garnettii																															ns	ns	ns	****	ns	*	**	ns	****	ns
Callithrix_jacchus																																ns	ns	ns	ns	ns	ns	ns	***	ns
Saimiri_boliviensis																																	ns	ns	ns	ns	ns	ns	***	ns
Macaca_mulatta																																		ns	ns	ns	ns	ns	ns	ns
Papio_anubis																																			ns	ns	ns	ns	ns	ns
Nomascus_leucogenys																																				ns	ns	ns	*	ns
Pongo_abelii																																					ns	ns	ns	ns
Gorilla_gorilla																																						ns	ns	ns
Pan_paniscus																																							**	ns
Pan_troglodytes																																								***
