# Supplementary material for: Evolutionary conservation in genes underlying human psychiatric disorders
Source: Front Hum Neurosci. 2014 May 6;8:283. doi: 10.3389/fnhum.2014.00283 (PMC4018557; doi:10.3389/fnhum.2014.00283)

SFARI autism genes scored

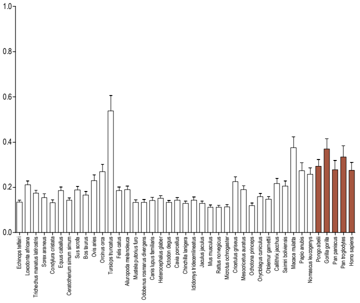


SFARI autism genes non-syndromic 5+

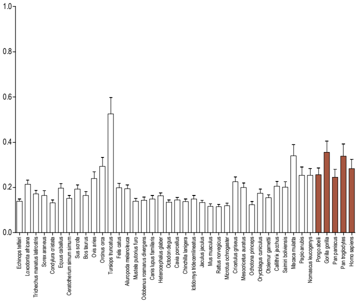


SFARI autism genes non-syndromic 4+

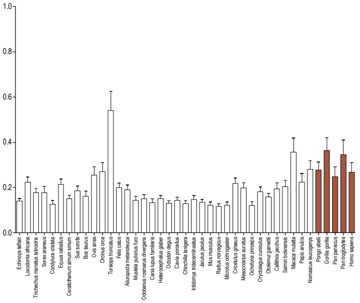


SFARI autism genes non-syndromic 3+

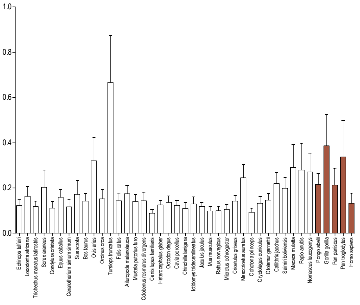

Supplement: Supplementary file 5 [file DataSheet3.PDF]
